# Supplementary material for: Redox-responsive polymer micelles co-encapsulating immune checkpoint inhibitors and chemotherapeutic agents for glioblastoma therapy
Source: Nat Commun. 2024 Feb 6;15:1118. doi: 10.1038/s41467-024-44963-3 (PMC10847518; doi:10.1038/s41467-024-44963-3)
Supplement: Supplementary file 1 — Supplementary Information [file 41467_2024_44963_MOESM1_ESM.pdf]

Supplementary Information for

Redox-responsive polymer micelles co-encapsulating immune checkpoint inhibitors and chemotherapeutic agents for glioblastoma therapy

Zhiqi Zhang[1], Xiaoxuan Xu[1], Jiawei Du[1], Xin Chen[2], Yonger Xue[3], Jianqiong Zhang[1,2], Xue Yang[1], Xiaoyuan Chen[4,5,6,7*], Jinbing Xie[1*]and Shenghong Ju[1*]

[1]Jiangsu Key Laboratory of Molecular and Functional Imaging, Department of Radiology, Zhongda Hospital, Medical School, Southeast University, Nanjing, 210009, China.

[2]Department of Microbiology and Immunology, Medical School, Southeast University, Nanjing, 210009, China.

[3]Center for BioDelivery Sciences, School of Pharmacy, Shanghai Jiao Tong University, Shanghai, 200240, China.

[4]Departments of Diagnostic Radiology, Surgery, Chemical and Biomolecular Engineering, and Biomedical Engineering, Yong Loo Lin School of Medicine and College of Design and Engineering, National University of Singapore, Singapore, 119074, Singapore.

[5]Nanomedicine Translational Research Program, NUS Center for Nanomedicine, Yong Loo Lin School of Medicine, National University of Singapore, Singapore, 117597, Singapore.

[6]Clinical Imaging Research Centre, Centre for Translational Medicine, Yong Loo Lin School of Medicine, National University of Singapore, Singapore, 117599, Singapore

[7]Institute of Molecular and Cell Biology, Agency for Science, Technology, and Research (A*STAR), 61 Biopolis Drive, Proteos, Singapore, 138673, Singapore.

*Correspondence to: jsh@seu.edu.cn (S.J.); chen.shawn@nus.edu.sg (X.C);

xiejb@seu.edu.cn (J.X.)

The PDF file includes:

Supplementary Figures 1 to 48

Supplementary Tables 1 to 2

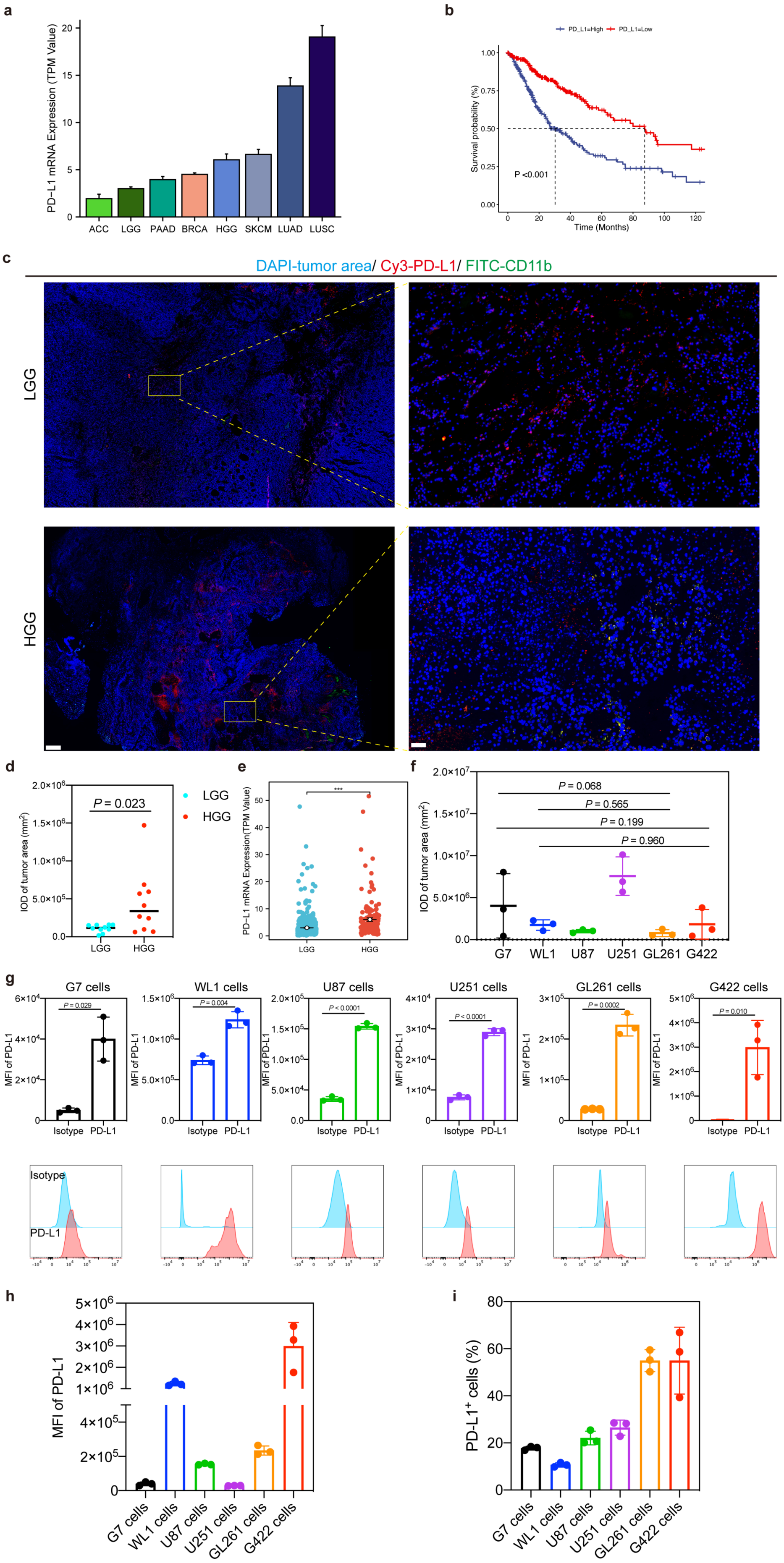
a
PD-L1 mRNA Expression (TPM Value)
ACC LGG PAAD BRCA HGG SKCM LUAD LUSC
b
PD_L1=High PD_L1=Low
Survival probability (%)
P <0.001
Time (Months)
c
DAPI-tumor area/ Cy3-PD-L1/ FITC-CD11b
LGG
HGG
d
IOD of tumor area (mm2)
P = 0.023
LGG
HGG
e
PD-L1 mRNA Expression(TPM Value)
***
f
IOD of tumor area (mm2)
P = 0.068
P = 0.565
P = 0.199
P = 0.960
G7 WL1 U87 U251 GL261 G422
g
G7 cells
WL1 cells
U87 cells
U251 cells
GL261 cells
G422 cells
MFI of PD-L1
P = 0.029
P = 0.004
P < 0.0001
P < 0.0001
P = 0.0002
P = 0.010
Isotype PD-L1
Isotype
PD-L1
h
MFI of PD-L1
i
PD-L1+ cells (%)
G7 cells WL1 cells U87 cells U251 cells GL261 cells G422 cells

34

Suppl

ementary Figure 1. PD-L1 expression in GBM patients and tumor cell bearing-mice models. a PD-L1 expression percentage in clinical patients (LGG, n = 500; HGG, n = 144). **b** Survival curves of glioma patients from TCGA database (LGG, n = 636; HGG, n = 138) (PD-L1 expression ≥ 50% was defined as high expression, < 50% was defined as low expression). **c, d** Respective images and qualification of PD-L1 immunofluorescence of LGG (n = 10) and HGG (n = 10) histological sections obtained from patients. The left images scale bar = 500 μm, the right images scale bar = 50 μm. We only counted the Cy3-PD-L1 fluorescence and excluded the overlap of PD-L1 with CD11b. **e** PD-L1 analysis of 144 HGG and 500 LGG cases acquired from TCGA database. Each dot represented a single individual. **f** Comparison of PD-L1 immunofluorescence of the histological sections of G7, WL1, U87, U251, GL261 and G422 tumor-bearing mice. We only counted the Cy3-PD-L1 fluorescence and excluded the overlap of PD-L1 with CD11b, n = 3. **g** Representative flow cytometry histogram and qualification of PD-L1 expression on G7, WL1, U87, U251, GL261 and G422 cells, n = 3. **h, i** MFI and percentage of PD-L1 expression G7, WL1, U87, U251, GL261 and G422 cells, n = 3. HGG, high grade glioma; LGG, low grade glioma; LUSC, lung squamous cell carcinoma, n = 496; LUAD, lung adenocarcinoma, n = 510; SKCM, skin cutaneous melanoma, n = 469; BRCA, breast invasive carcinoma, n = 1079; PAAD, pancreatic adenocarcinoma, n = 178; ACC, adrenocortical carcinoma, n = 79, the numbers of patients indicated in the legend for LUSC, LUAD, SKCM, BRCA, PAAD, and ACC apply to **a**. TPM, Transcripts Per Million. G7 and WL1 cells were GBM patient-derived xenograft (PDX) cell lines, U87 and U251 cells were human glioma cell lines, GL261 and G422 cells were mice glioma cell lines. ******P* <0.001, statistical significance was calculated by one-way ANOVA with Fisher's LSD test in (f), and independent sample *t-test* in (d) and (g).

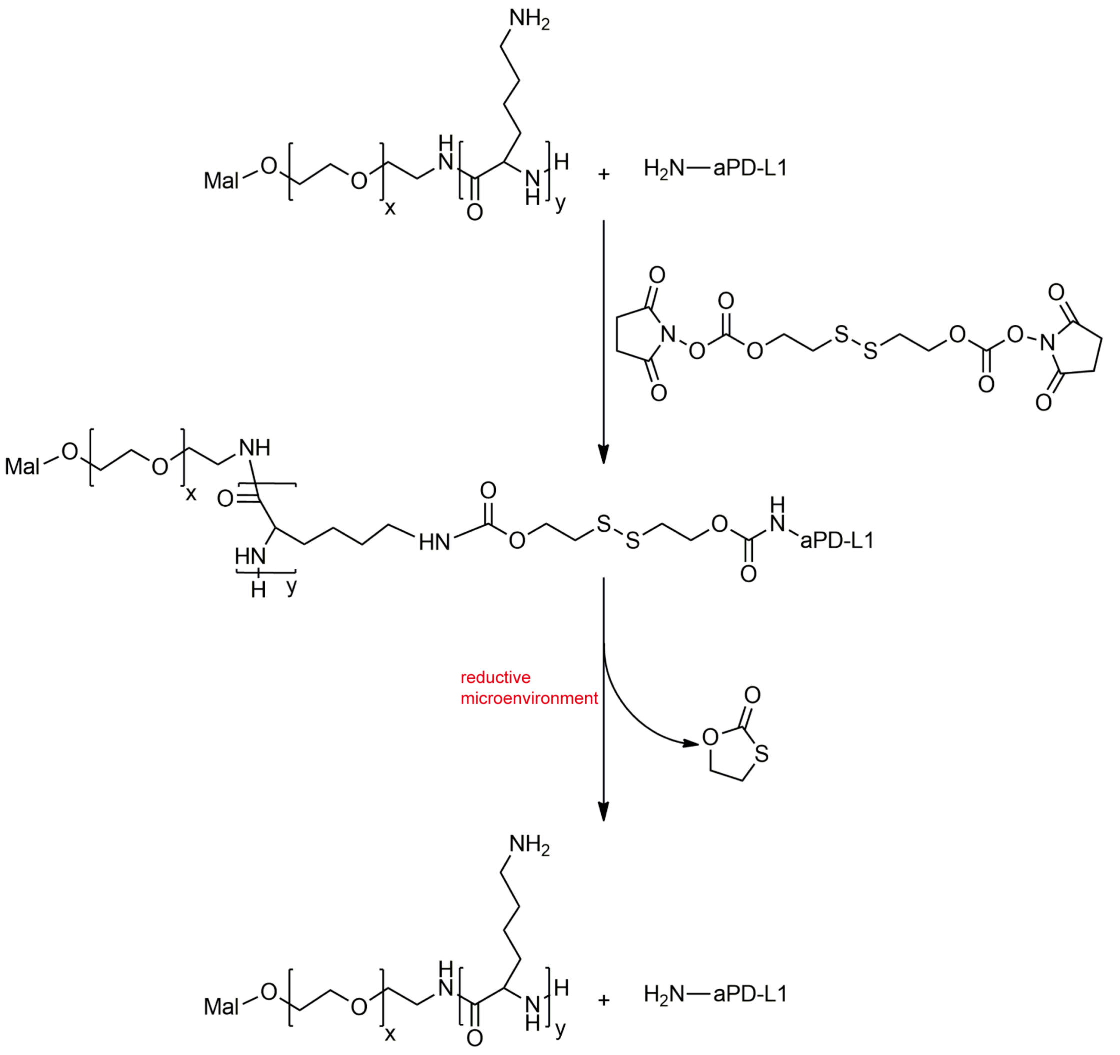

Supplementary Figure 2. Scheme for the synthesis and reduction-induced detachment of Mal-PEG-PLL, SC-(CH$_2$)$_2$-S-S-(CH$_2$)$_2$-SC and aPD-L1. Scheme for Mal-PEG-PLL, SC-(CH$_2$)$_2$-S-S-(CH$_2$)$_2$-SC and aPD-L1 synthesis and for release of aPD-L1 in response to reductive microenvironment.

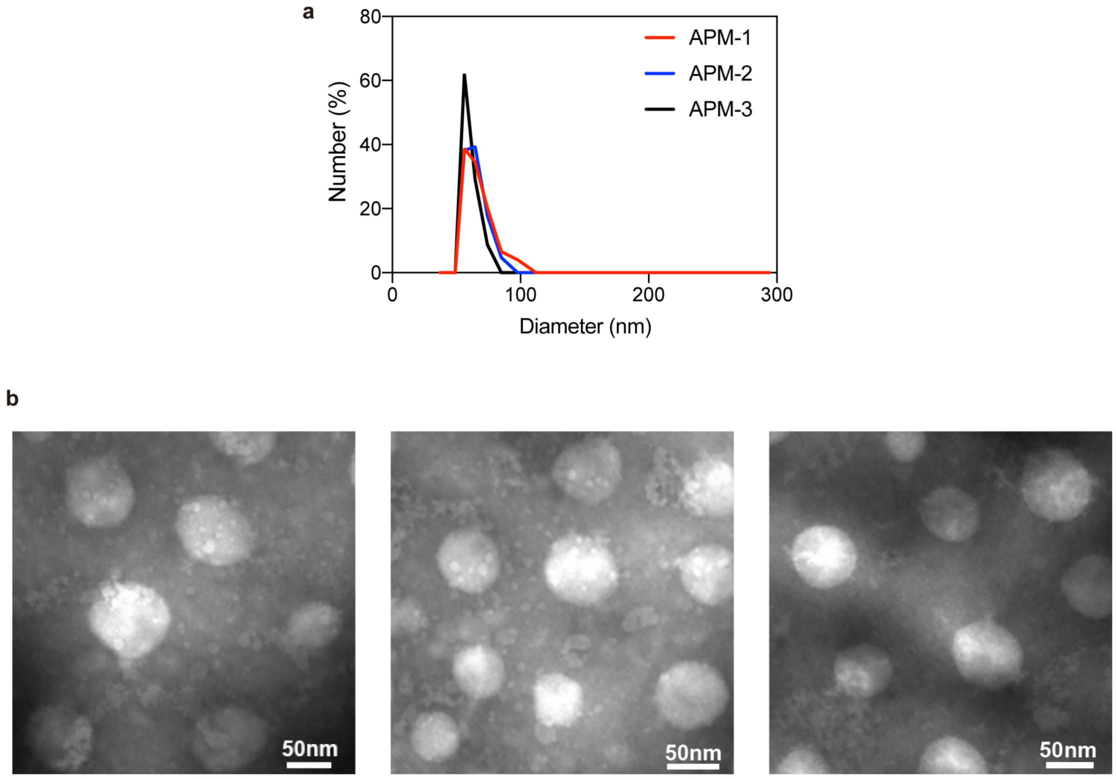

Supplementary Figure 3. Size distribution and morphology of APM by DLS and TEM a DLS analysis of the mean particle size distribution of APM. **b** TEM image of APM. Scale bar = 50 nm. n = 3 biologically independent samples.

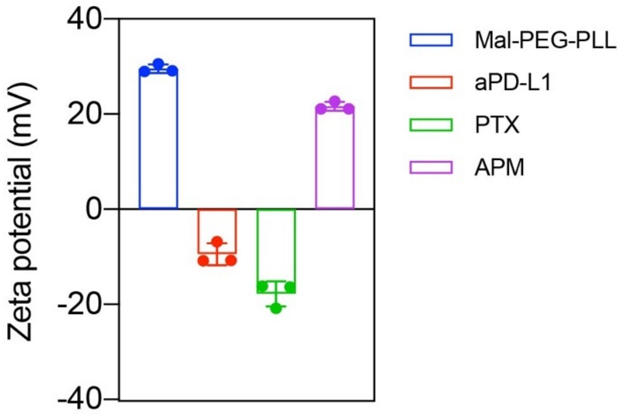

Supplementary Figure 4. Surface potential characterization of Mal-PEG-PLL, free aPD-L1, free PTX and APM. Zeta potential of Mal-PEG-PLL, free aPD-L1, free PTX and APM. n = 3 biologically independent samples, data are presented as mean ± SD.

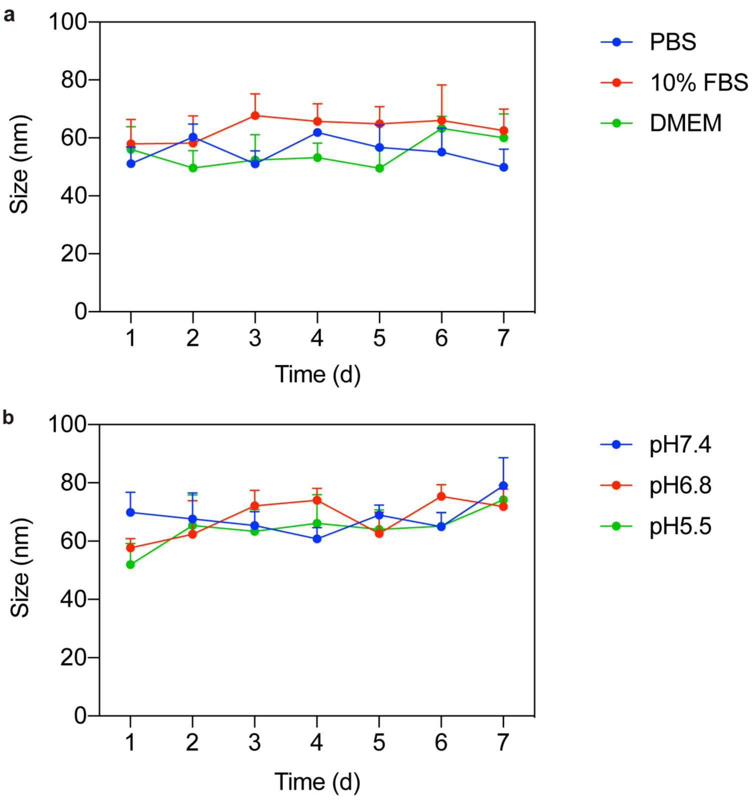

Supplementary Figure 5. Stability characteristics of APM. a. DLS analysis of APM was measured in PBS (pH = 7.4), DMEM medium and 10% FBS for seven consecutive days, respectively. **b.** DLS analysis of APM was measured in pH7.4 PBS, pH6.8 PBS and pH5.5 PBS for seven consecutive days, respectively. n = 3 biologically independent samples, data are presented as means ± SD.

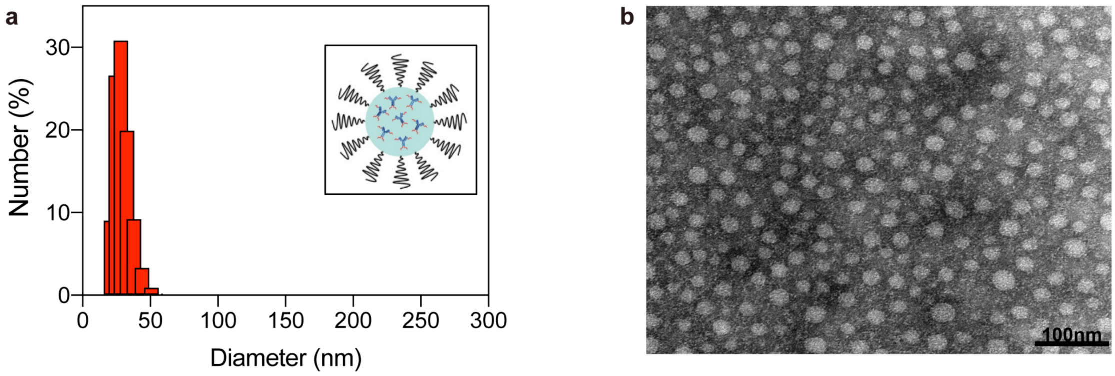

Supplementary Figure 6. Characterization of AM. a. Representative DLS analysis of the average particle size distribution of AM. Inset: schematic illustration of AM. **b.** Representative TEM image of AM. Scale bar = 100 nm. The experiment was conducted independently three times with similar results. BioRender.com was used to create inset of (a)

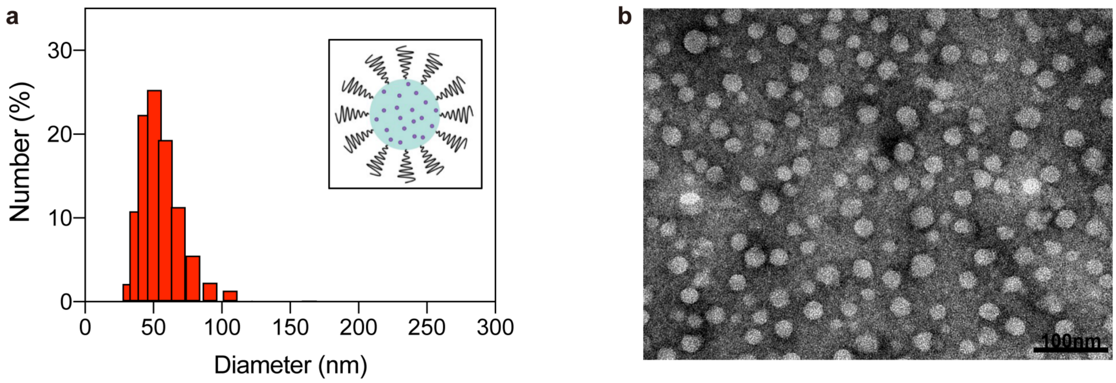

Supplementary Figure 7. Characterization of PM. a. Representative DLS analysis of the average particle size distribution of PM. Inset: schematic illustration of PM. **b.** Representative TEM image of PM. Scale bar = 100 nm. The experiment was conducted independently three times with similar results. BioRender.com was used to create inset of (a)

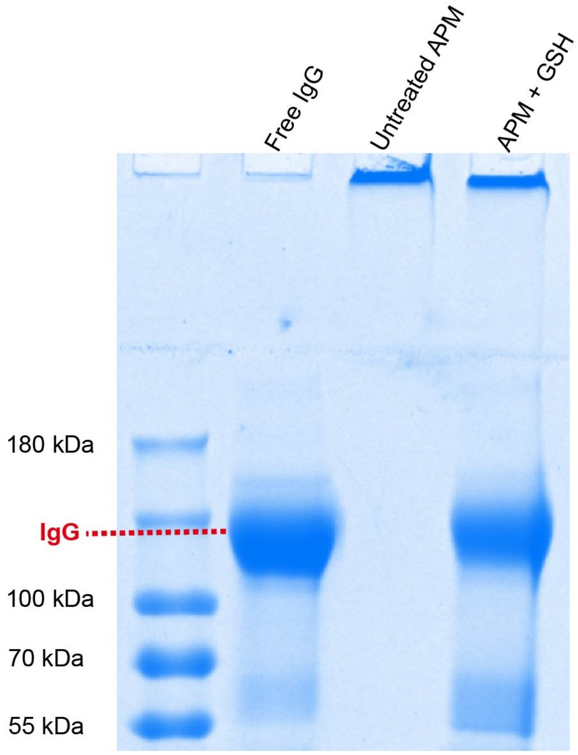

Supplementary Figure 8. Reductive-induced recovery of IgG structure from APM. Representative image of SDS–polyacrylamide gel electrophoresis (PAGE) picture of APM pretreated with or without GSH (7 μg of aPD-L1 per sample). The experiment was conducted independently twice with similar results.

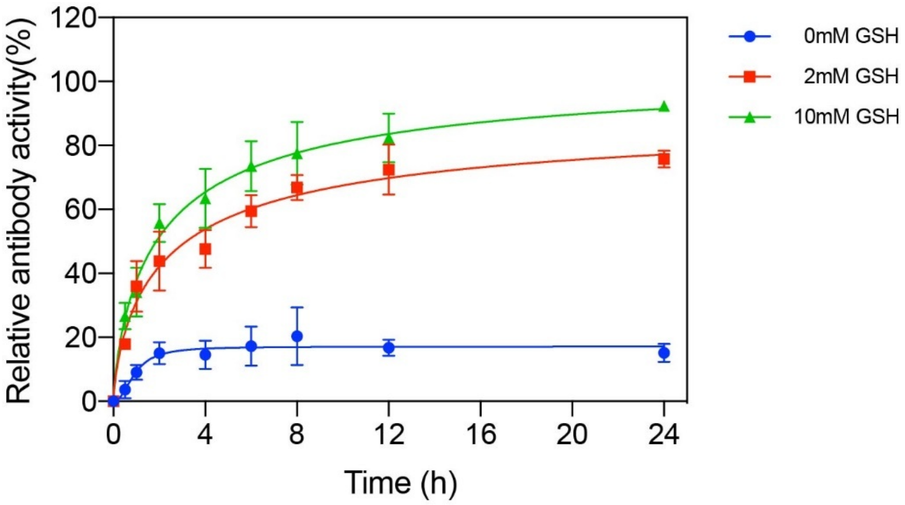

Supplementary Figure 9. Profiles of antibody release of APM under GSH treatment. Relative antibody activity towards IgG of APM incubated with different concentrations of GSH solution against time. n = 3 biologically independent samples, data are expressed as mean ±SD.

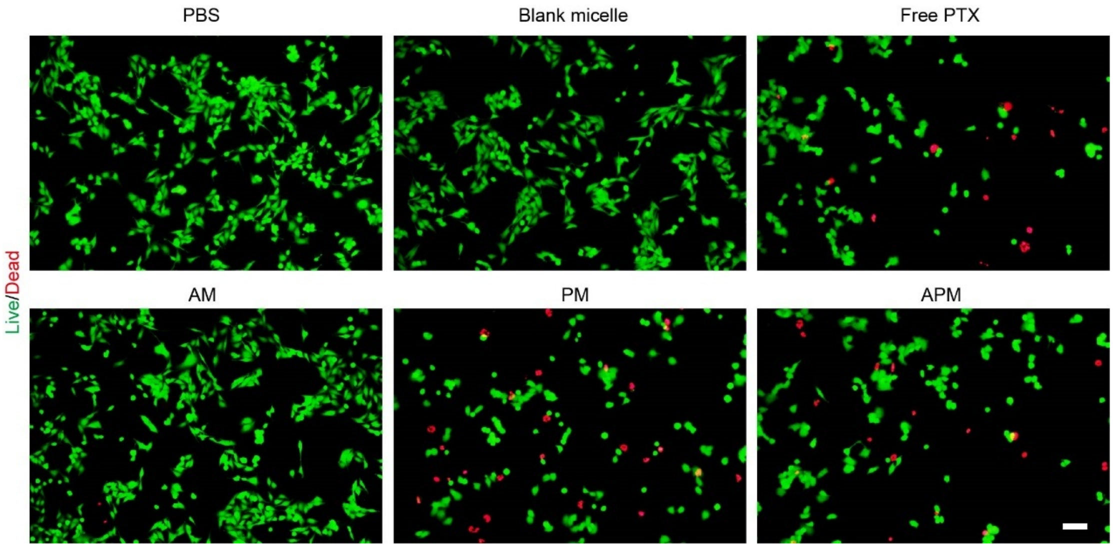

Supplementary Figure 10. Cytotoxicity of APM. After 24 h of co-cultivation with different formulas, the GL261 cells were stained with Calcein/PI Live/Dead Viability/Cytotoxicity assay kit. Green: live cells; red: dead cells; scale bar = 100 μm. The experiment was conducted independently three times with similar results.

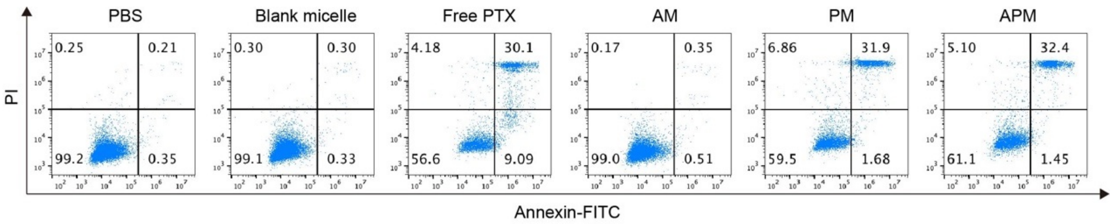

Supplementary Figure 11. Apoptosis induced by APM. Representative flow cytometry dot plots of apoptosis results of GL261 cells after incubation with different formulas for 24 h. n = 3 biologically independent samples.

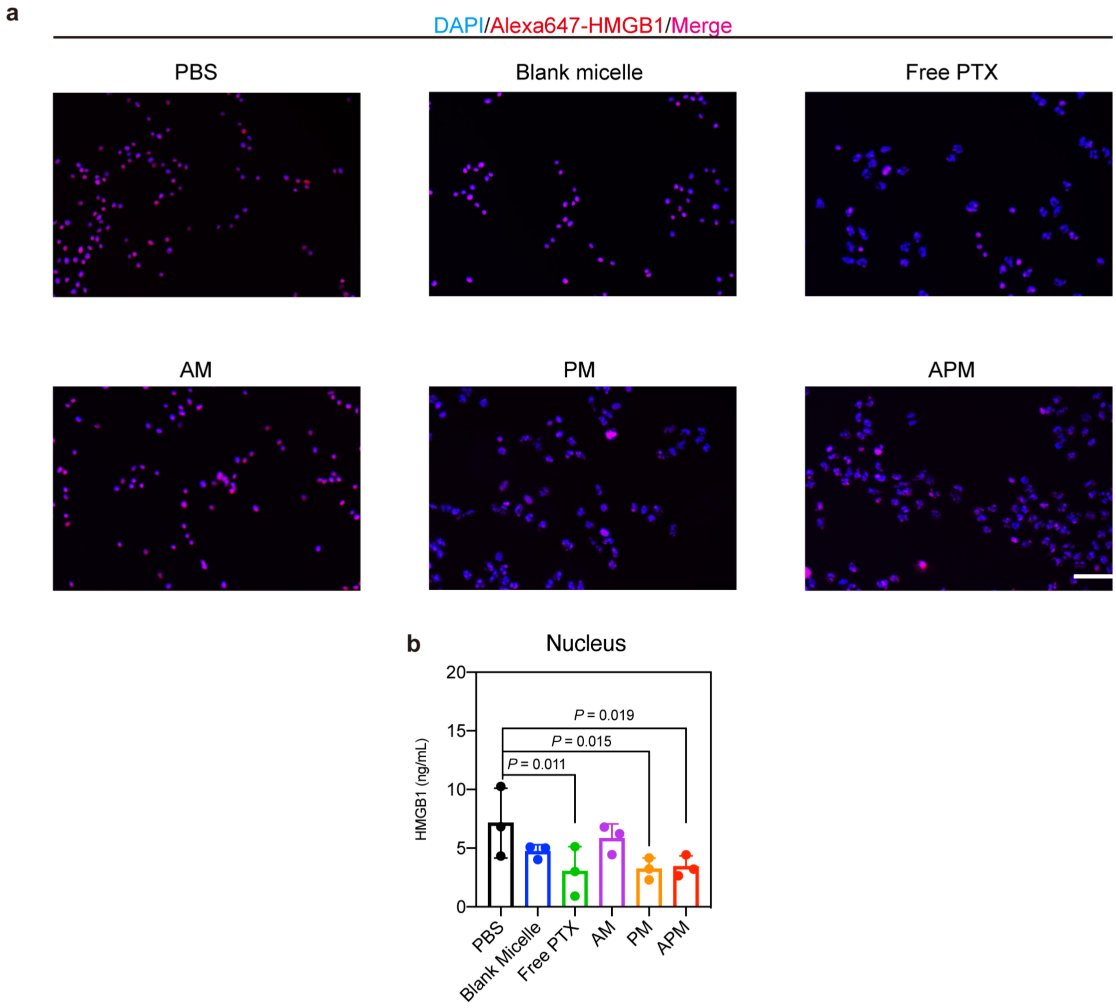

Supplementary Figure 12. Intracellular HMGB1 levels after different treatments. a, b After 24 h of co-cultivation with different formulas, the HMGB1 was observed with a fluorescence microscope and the level of intracellular HMGB1 after receiving different treatments. Scale bar = 50 μm. The experiment was conducted independently three times with similar results. Statistical significance was calculated by one-way ANOVA with Fisher's LSD test.

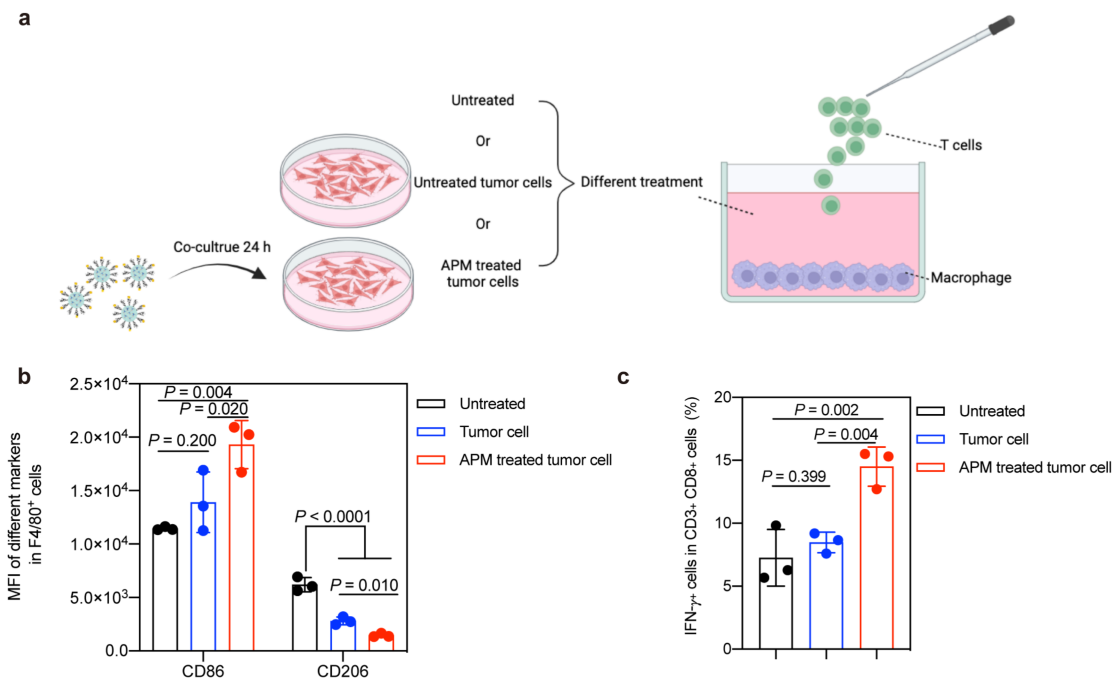

Supplementary Figure 13. APM shifts macrophages toward a pro-inflammatory phenotype and enhanced T cell function. a Schematic illustration of the *in vitro* model to evaluate the effect of untreated tumor cells and APM treated tumor cells on macrophages and T cells. **b** MFI of $CD86^+$ and $CD206^+$ cells in $F4/80^+CD11b^+$ cells by flow cytometry. **c** Percentage of $IFN\gamma^+$ cells in $CD3^+CD8^+$ T cells by flow cytometry. n = 3. Statistical significance was calculated by one-way ANOVA with Fisher's LSD test. BioRender.com was used to create (a)

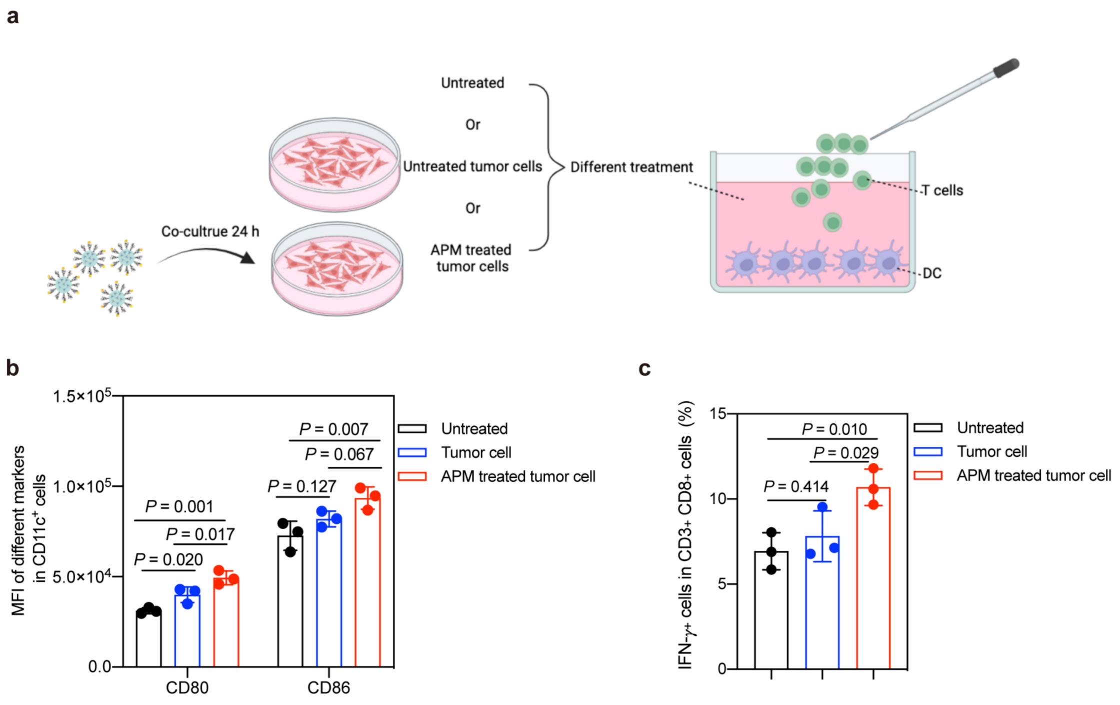

Supplementary Figure 14. APM promotes DC maturation and activates T cell function. a Schematic illustration of the *in vitro* model to evaluate the effect of untreated tumor cells and APM treated tumor cells on DC maturation and mDC-induced T cell activation. **b** MFI of $CD80^+$ and $CD86^+$ cells in $CD11c^+$ cells by flow cytometry. **c** Percentage of $IFN\gamma^+$ cells in $CD3^+CD8^+$ T cells by flow cytometry. n = 3. Statistical significance was calculated by one-way ANOVA with Fisher's LSD test. BioRender.com was used to create (a)

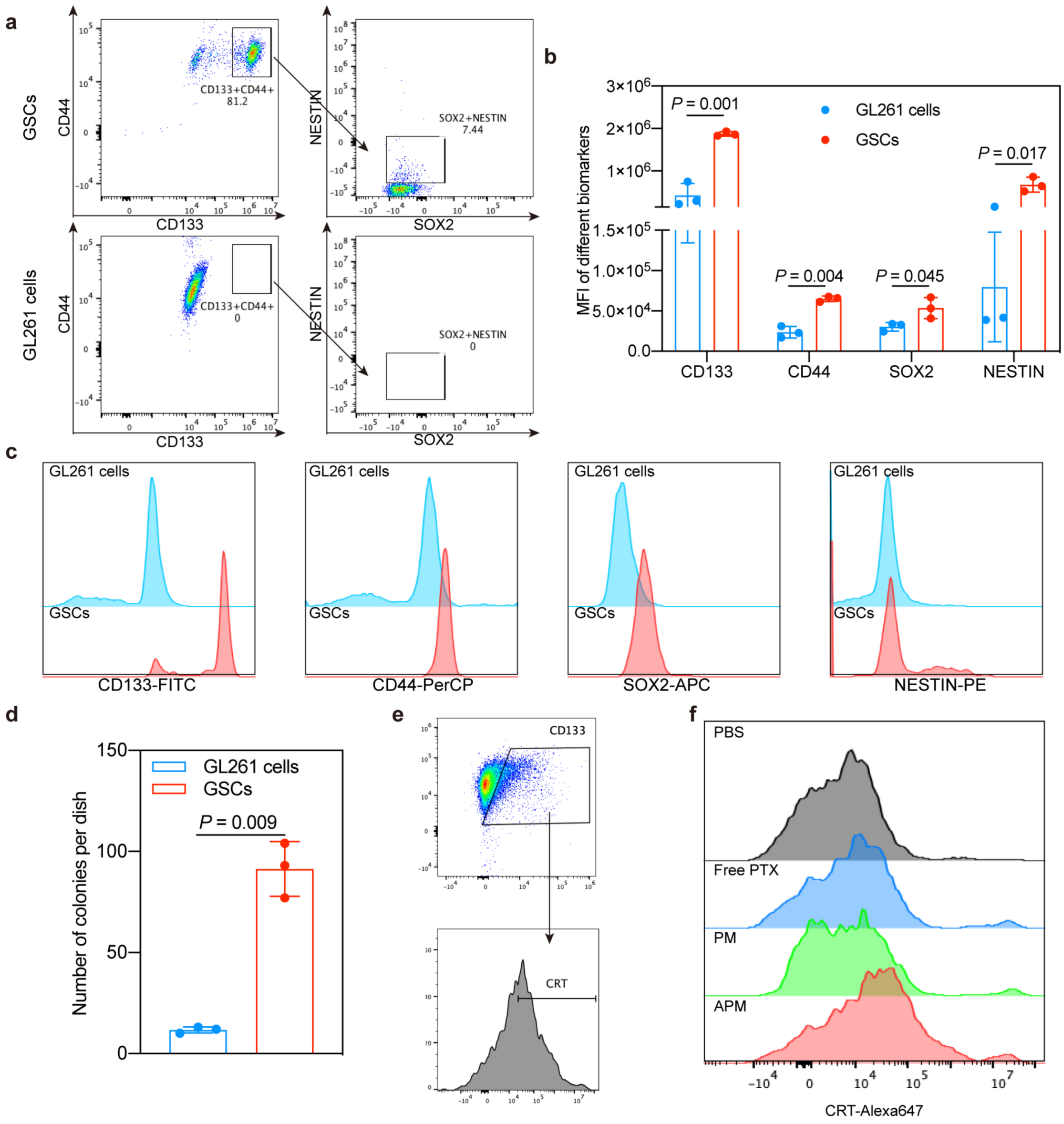

Supplementary Figure 15. Identification of glioma stem-like cells and the *in vitro* effect of APM on glioma stem-like cells. a Gating strategy to sort SOX2$^+$NESTIN$^+$ cells in glioma stem-like cells (GSCs) or GL261 cells gating on CD133$^+$ CD44$^+$ cells. **b**, **c** Qualification and representative flow cytometry histogram of the expression of CD133, CD44, SOX2, NESTIN on GL261 cells or GSCs. **d** Statistical analysis of soft agar assays of GL261 cells and GSCs. Colonies > 0.5 mm were counted. **e** Gating strategy to sort CRT$^+$ cells in GSCs gating on CD133$^+$ cells. **f** Representative flow cytometry histogram of the exposure of CRT on GSCs after receiving different treatments 24 hours. Data are presented as means ± SD, n = 3 biologically independent samples. Statistical significance was calculated by an independent

sample *t-test*.

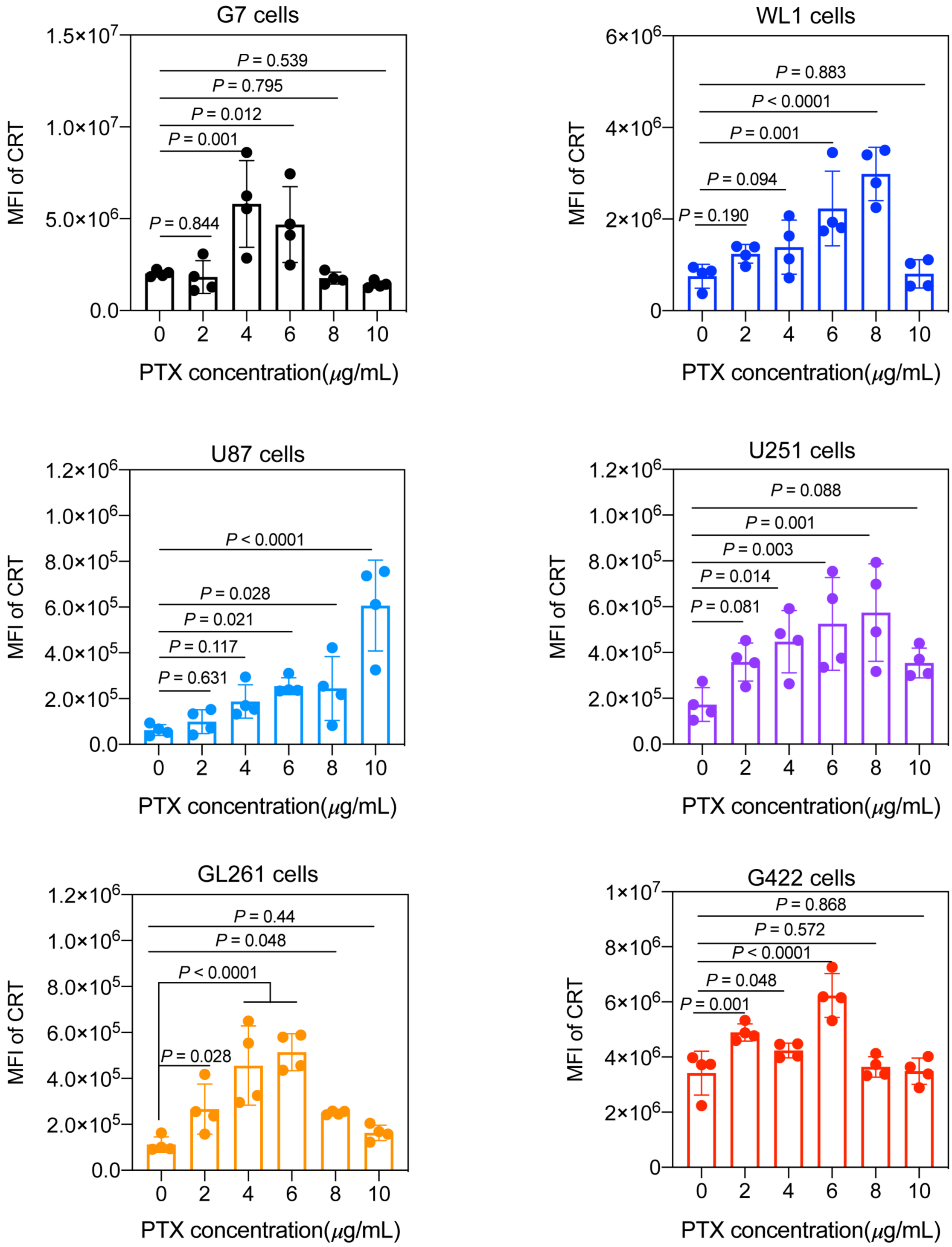

Supplementary Figure 16. Different concentrations of PTX induce different levels of CRT expression. Quantification of CRT exposure on the surface of G7, WL1, U87, U251, GL261 and G422 cells after receiving different PTX concentration treatments. n = 4 biologically independent samples. Statistical significance was

calculated by one-way ANOVA with Fisher’s LSD test.

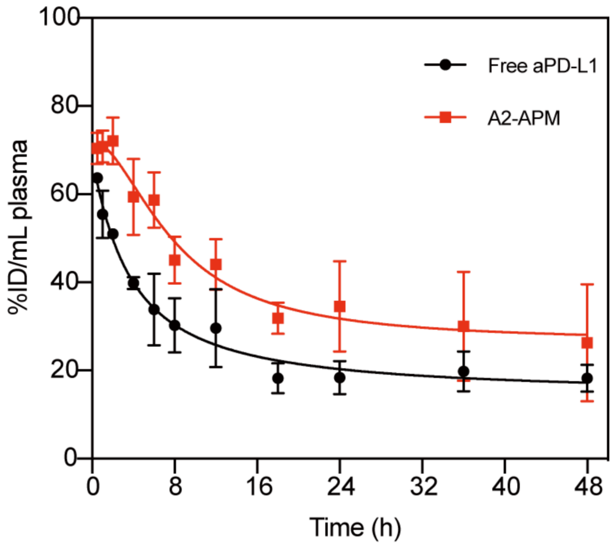

Supplementary Figure 17. Blood circulation of free aPD-L1 and A2-APM. *In vivo* pharmacokinetics after intravenous (i.v.) injection of rats with free aPD-L1, APM and A2-APM. n = 3. Data are presented as means ± SD.

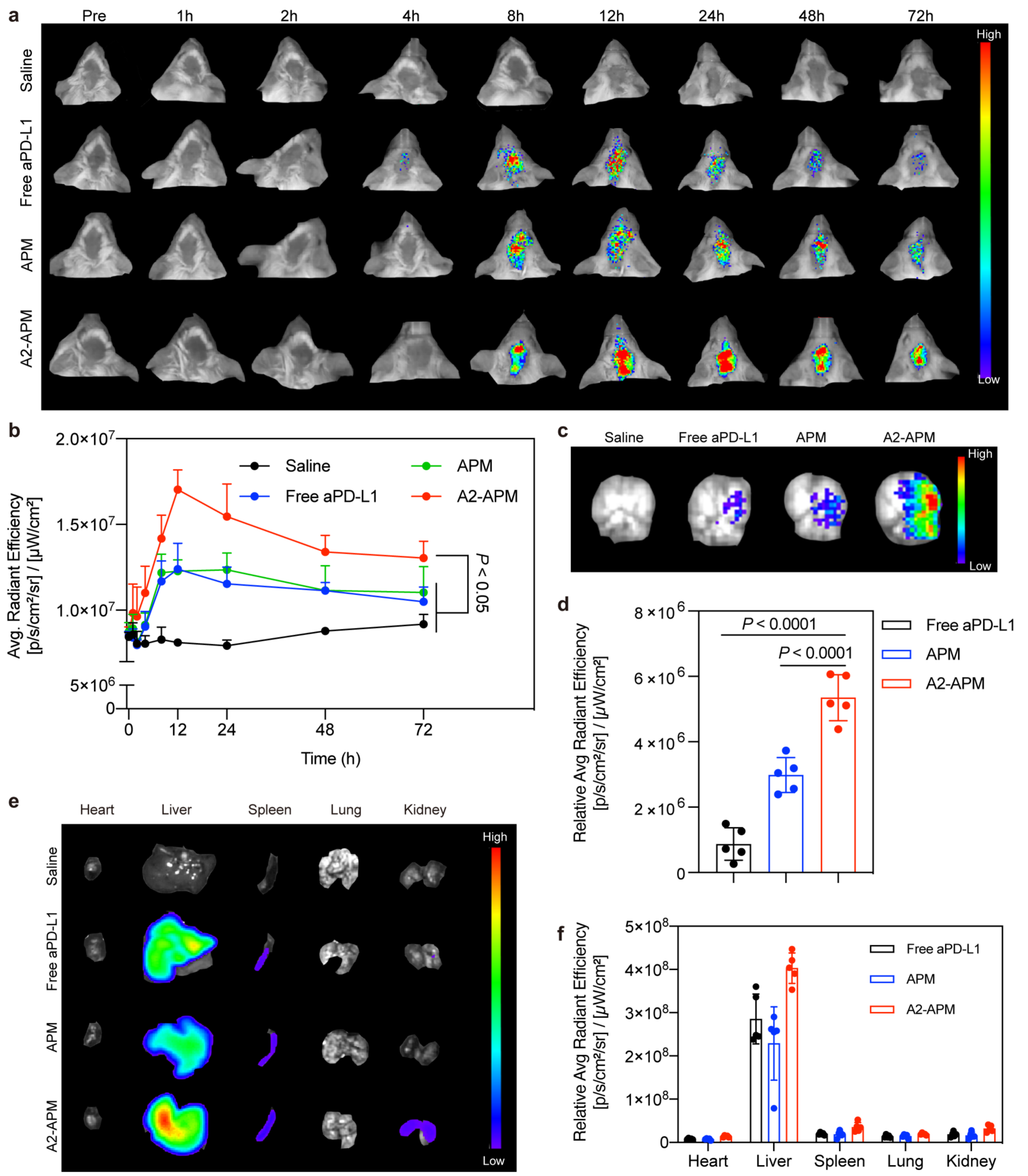

Supplementary Figure 18. Distribution of aPD-L1 in G422 GBM-bearing mice. a, b Fluorescence imaging and signal intensities at tumor sites at 1, 2, 4, 8, 12, 24, 48 and 72 h after i.v. injection of G422 tumor mice with saline, aPD-L1, APM and A2-APM. **c, d** Representative *ex vivo* fluorescence images and semiquantitative biodistribution of Cy7.5-aPD-L1 in brains collected from mice injected with saline, free aPD-L1, APM, A2-APM at 12 h post i.v. injection **e, f** Representative *ex vivo* fluorescence images and semiquantitative biodistribution of Cy7.5-aPD-L1 in major organs collected from mice injected with saline, free aPD-L1, APM, and A2-APM at

12 h post i.v. injection. n = 3 saline treated mice, n = 5 free aPD-L1, APM and A2-APM groups. All statistics are expressed as mean ± SD. Statistical significance was calculated by one-way ANOVA with Fisher's LSD test in (b) and (d).

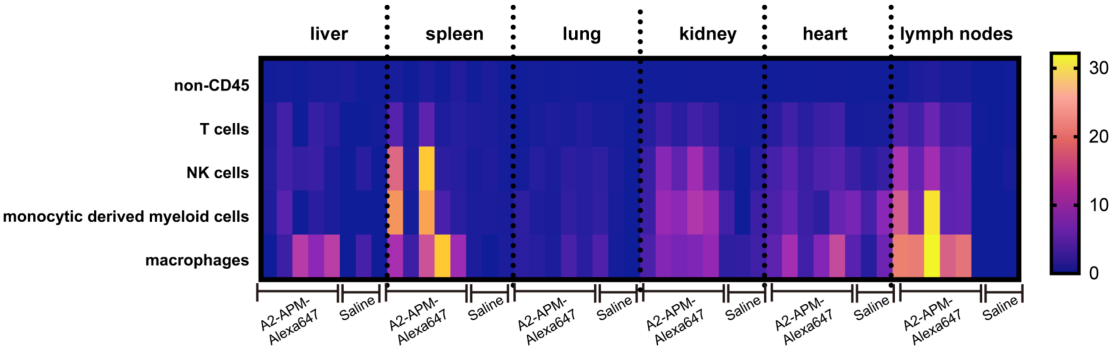

Supplementary Figure 19. Distribution of aPD-L1 in GL261 GBM-bearing mice by flow cytometry. Biodistribution analysis of A2-APM-Alexa647 at 90 min after a single, intravenous dose as assessed by flow cytometry of T cells (CD45$^+$CD3$^+$), NK cells (CD45$^+$NK1.1$^+$), microglia (CD45$^+$CD11b^{intermediate}), monocyte-derived myeloid cells (CD45$^+$CD11b^{high}Ly6C$^+$) and macrophages (CD45$^+$CD11b^{high}F4/80$^+$) in the liver, spleen, lung, kidneys, heart and lymph nodes. n = 5 A2-APM-Alexa647 treated mice and 3 saline-treated mice.

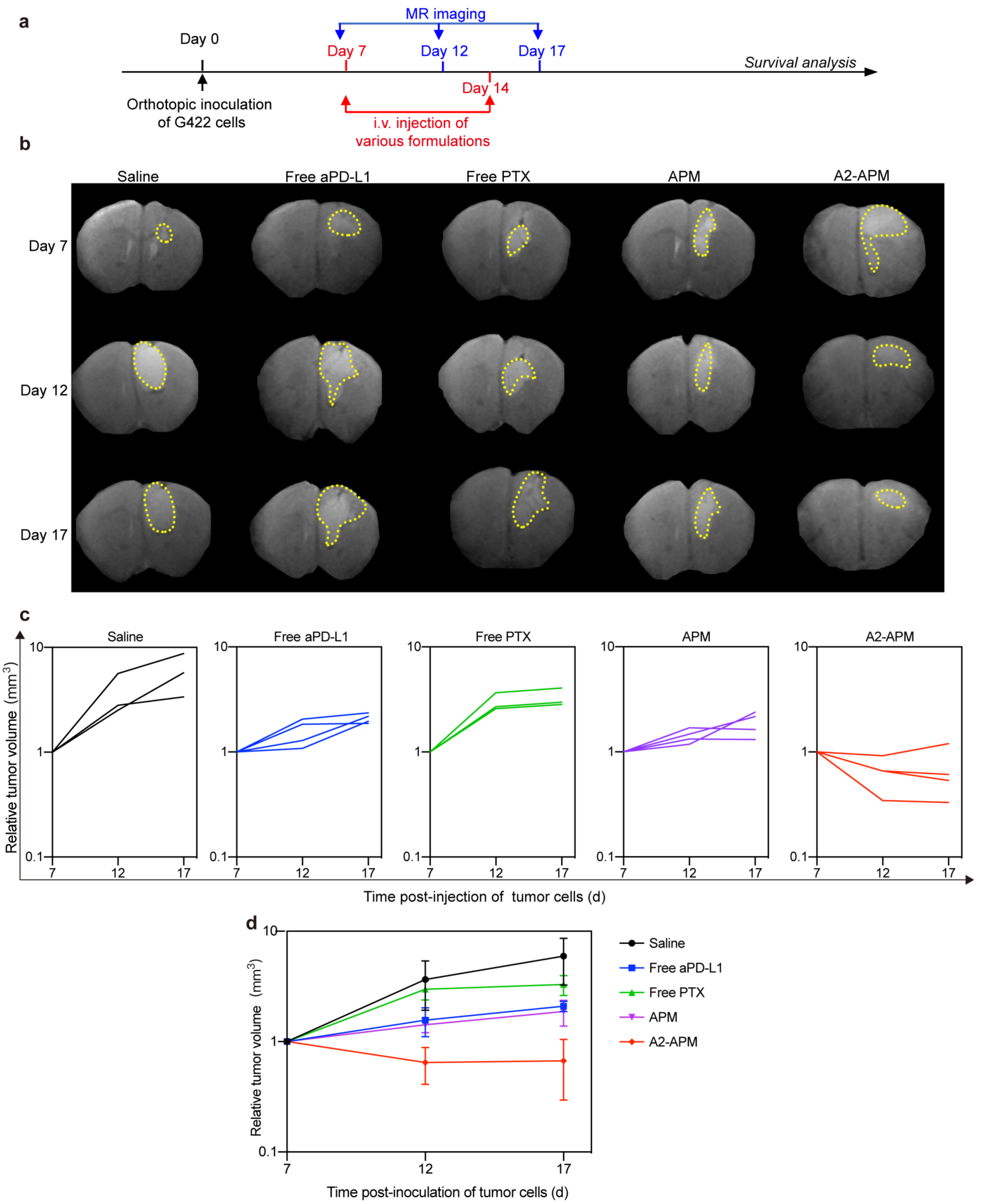

Supplementary Figure 20. *In vivo* evaluation of therapeutic efficacy of A2-APM in G422 GBM model. a Schematic of the treatment regimen. **b-d** Representative MR images and quantified tumor volumes of G422 tumor-bearing mice treated with saline, free aPD-L1, free PTX, APM, and A2-APM. The yellow dashed lines indicate the tumor area. n = 3 saline and free PTX treated mice, and 4 free aPD-L1, APM and A2-APM treated mice

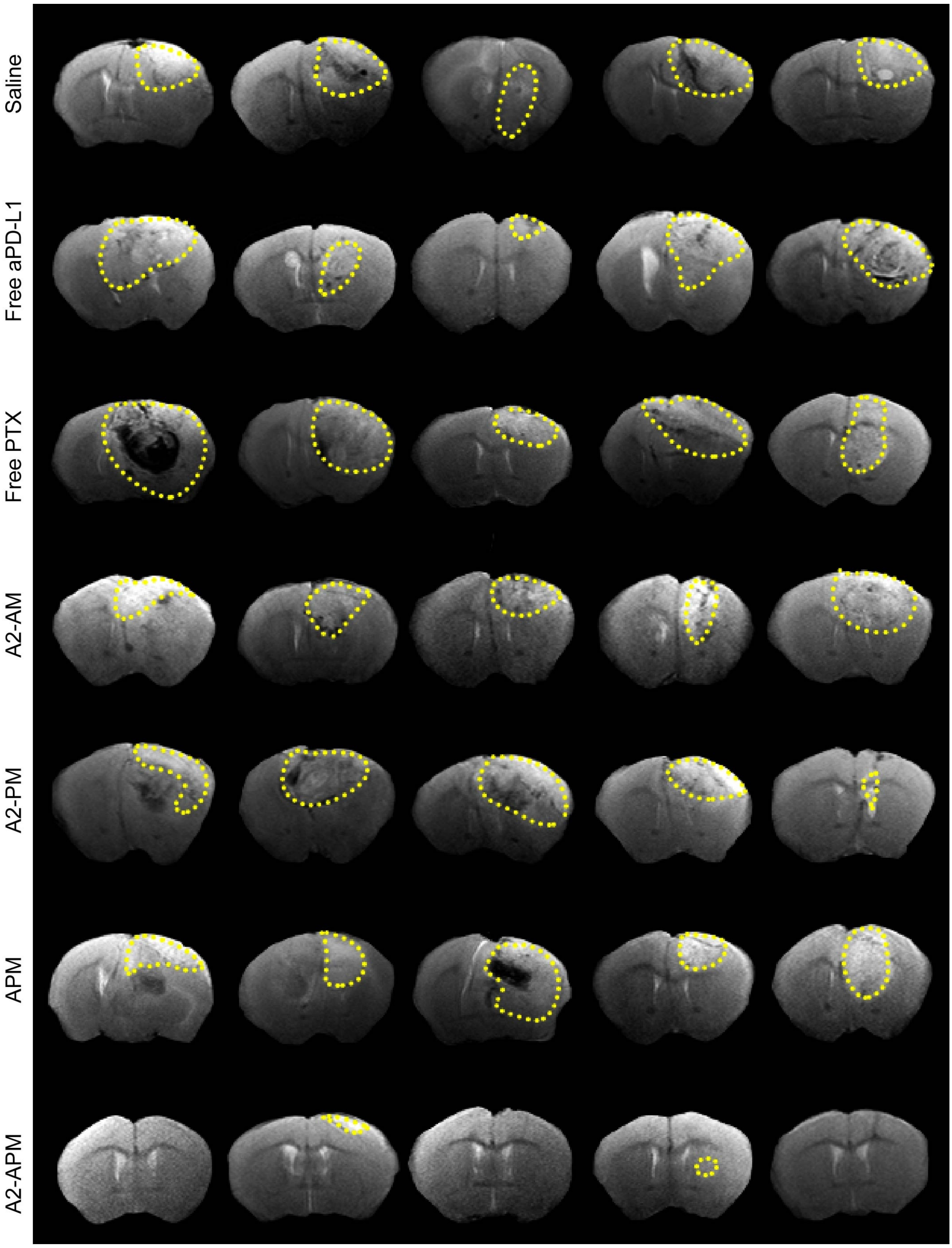

Supplementary Figure 21. MR images of the *in vivo* therapeutic effect of different treatments at day 22. MR images of GL261 tumor-bearing mice on day 22 after different treatments. n = 5. The yellow dashed lines indicate the tumor area.

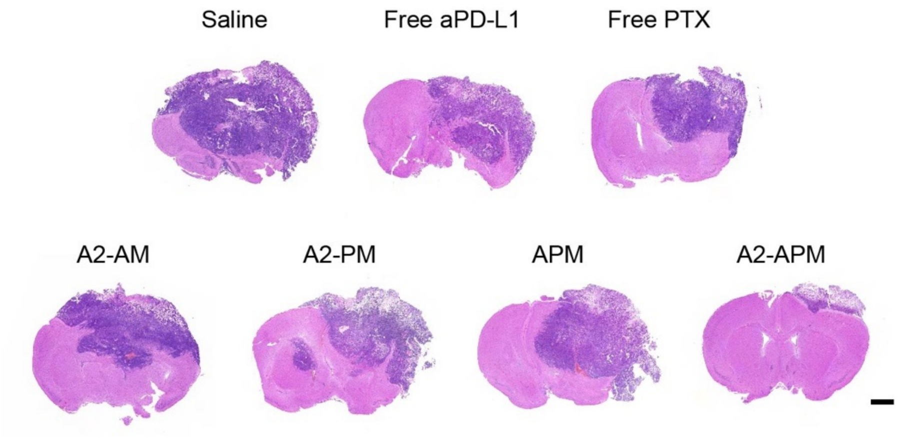

Supplementary Figure 22. H&E images of the tumor tissues after different treatments. Representative H&E images of GL261 tumor-bearing mice on day 30 after treatment with different formulas. Scale bar = 1000 μm. n = 3 biologically independent samples.

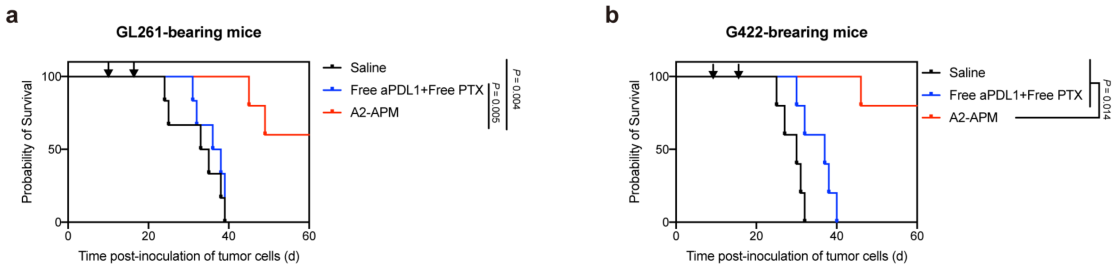

Supplementary Figure 23. Assessment of the additive effect of co-encapsulated PTX and aPD-L1delivery into the brain. a Survival curves for treated and control GL261 tumor mice. n = 6. **b** Survival curves for treated and control G422 tumor mice. n = 5. Statistical significance was calculated by log-rank test.

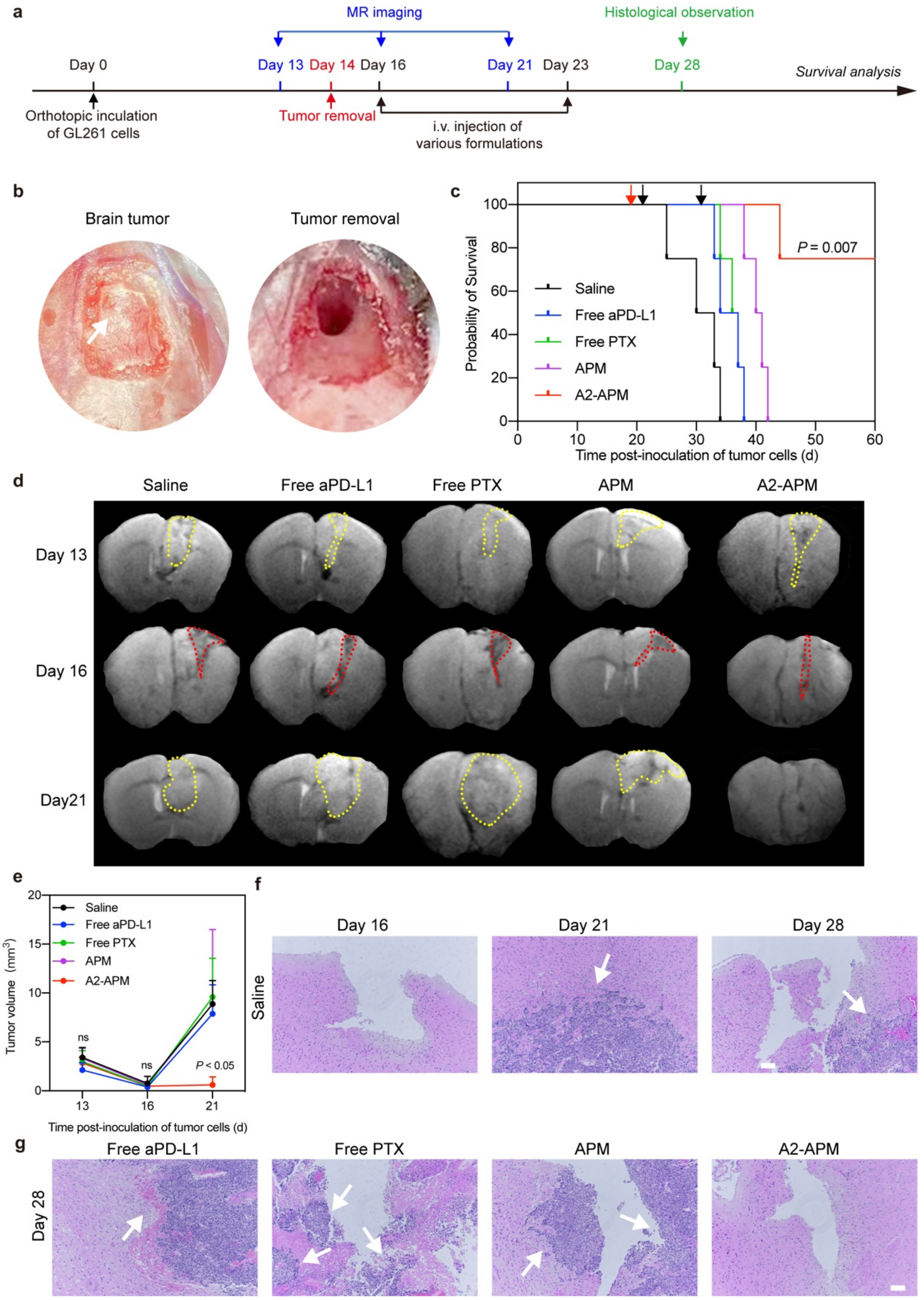

Supplementary Figure 24. A2-APM suppresses tumor recurrence of postresection GBM mice. a Schematic of the treatment regimen. **b** Surgery of the GL261 tumor-bearing mice on day 14 after tumor inoculation. The white arrow shows the tumor. **c** Survival curves for treated and control mice in postresection GBM mice.

n = 4, statistical significance was calculated by log-rank test. **d** Representative MR imaging of GL261 tumor-bearing mice treated with saline, free aPD-L1, free PTX, APM, and A2-APM. The yellow dashed lines indicate the tumor area, and the red dashed lines indicate the surgical cavity. n = 5. **e** MR quantification of tumor volume after GL261 tumor-bearing mice were treated with saline, free aPD-L1, free PTX, APM, and A2-APM, statistical significance was calculated by one-way ANOVA with Fisher's LSD test. **f** Representative H&E images of GL261 tumor-bearing mice treated with saline on days 16, 21, and 28. The white arrow shows invasive tumor cells and tumor cell islands. Scale bar = 100 µm. n = 3. **g** Representative H&E images of GL261 tumor-bearing mice treated with free aPD-L1, free PTX, APM, and A2-APM on day 28. The white arrow shows invasive tumor cells and tumor cell islands. Scale bar = 100 µm. n = 3.

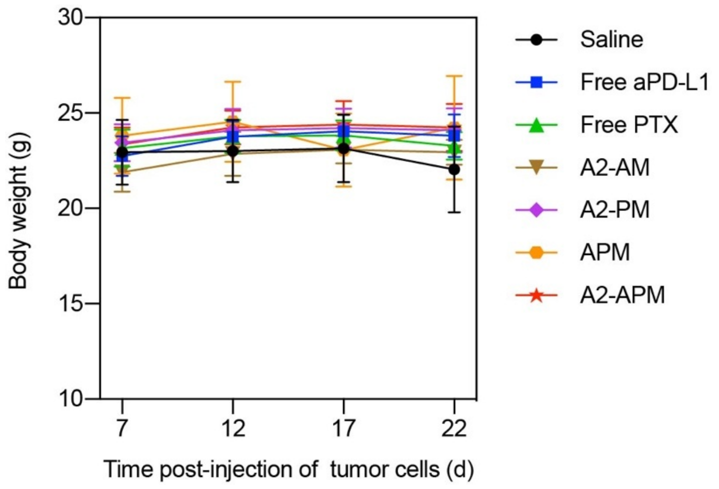

Supplementary Figure 25. Biosafety of different treatments in tumor bearing-mice with body weight. Body weight changes in GL261 tumor-bearing mice over 22 days treated with different formulas. n = 5, data are presented as mean ± SD.

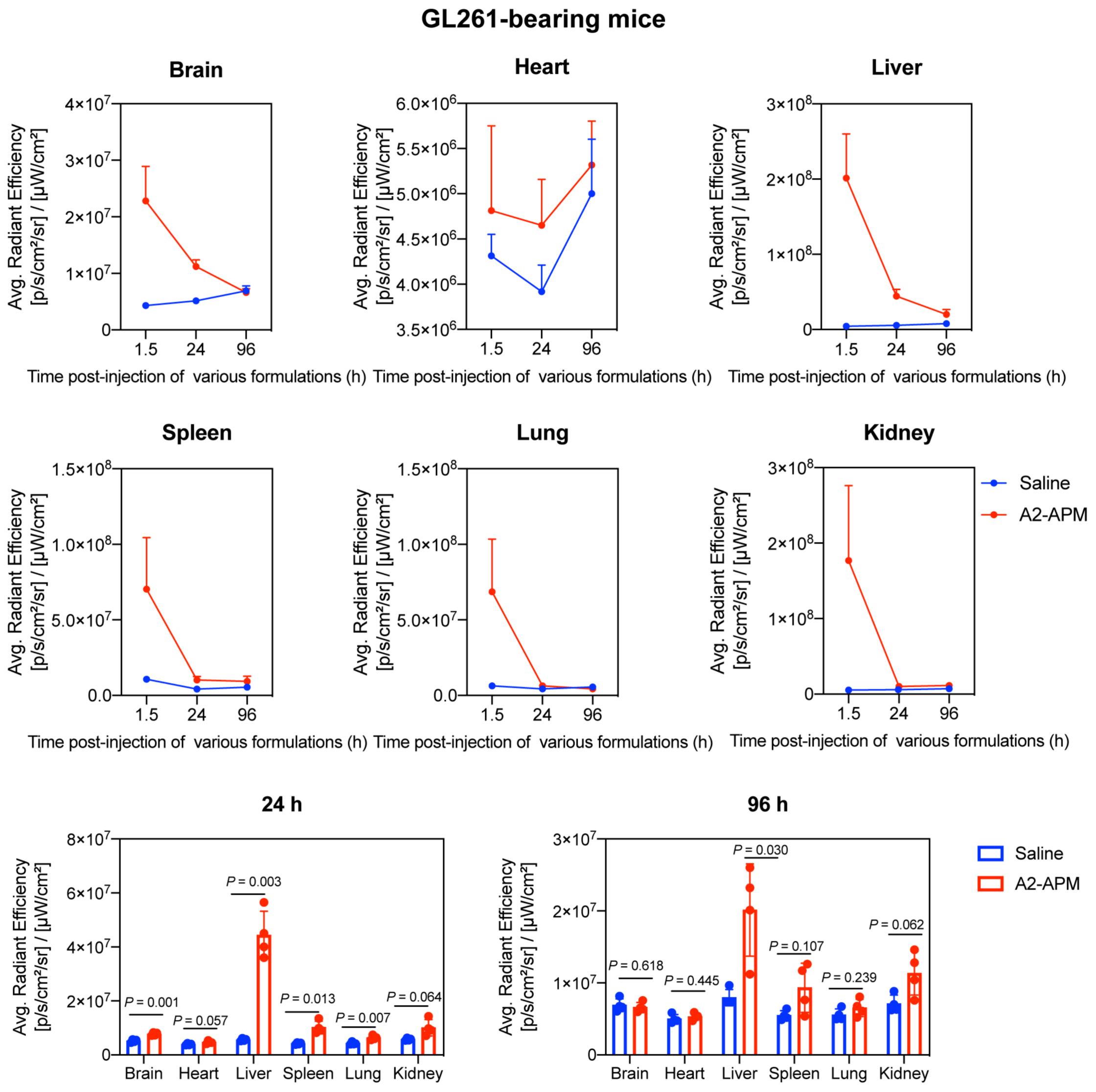
GL261-bearing mice
Brain
Heart
Liver
Spleen
Lung
Kidney
Avg. Radiant Efficiency [p/s/cm²/sr] / [μW/cm²]
Time post-injection of various formulations (h)
1.5
24
96
Saline
A2-APM
24 h
96 h
P = 0.001
P = 0.057
P = 0.003
P = 0.013
P = 0.007
P = 0.064
P = 0.618
P = 0.445
P = 0.030
P = 0.107
P = 0.239
P = 0.062

(Continued from previous page)

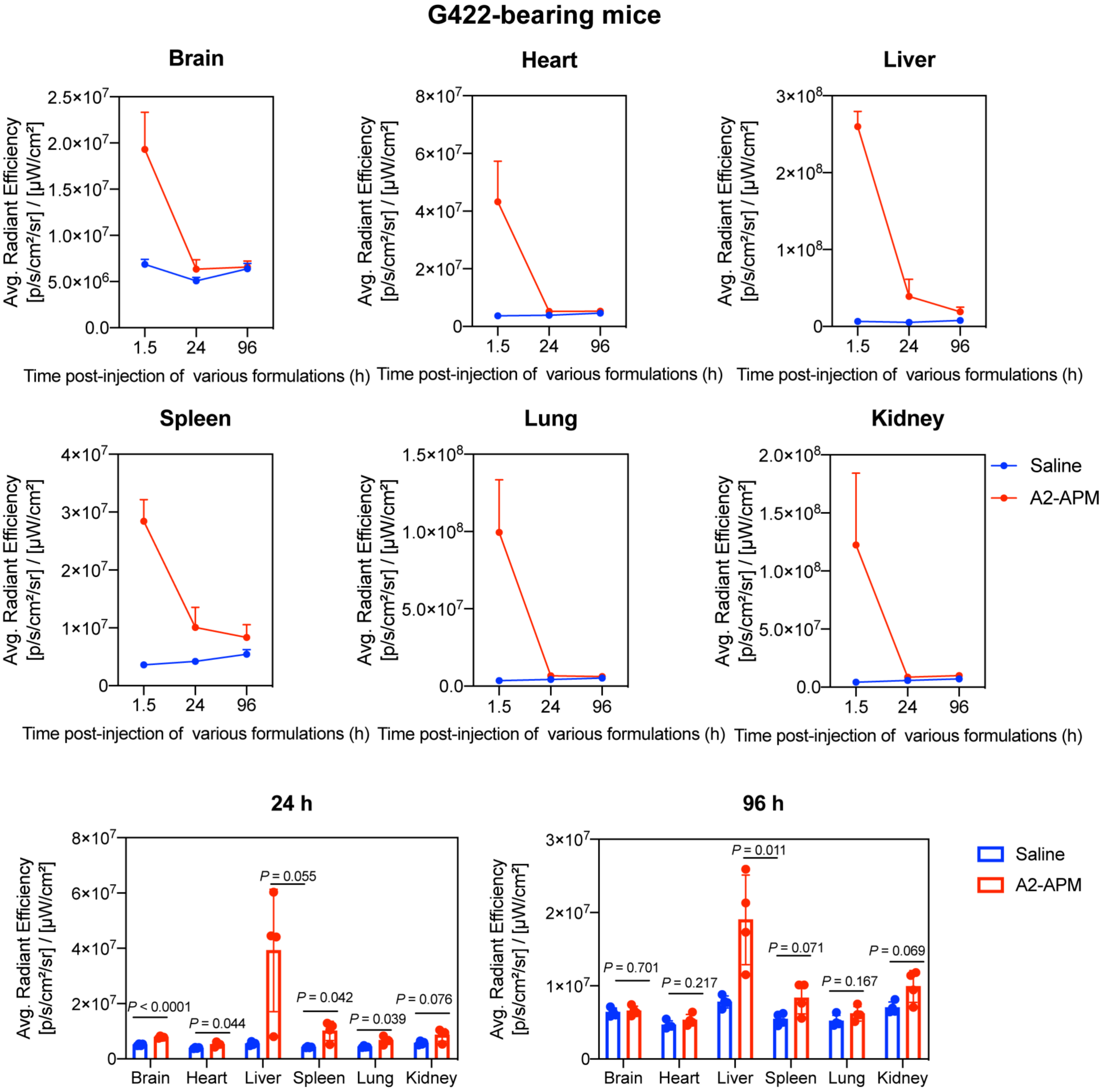

Supplementary Figure 26. A2-APM excretion from GBM mice over time. Semiquantitative biodistribution of Cy7.5-aPD-L1 in brain, heart, liver, spleen, lung, and kidneys collected from mice injected with saline and A2-APM at 1.5 h, 24 h and 96 h post *i.v.* injection (n = 4). Statistical significance was calculated by two-sided *t-test*.

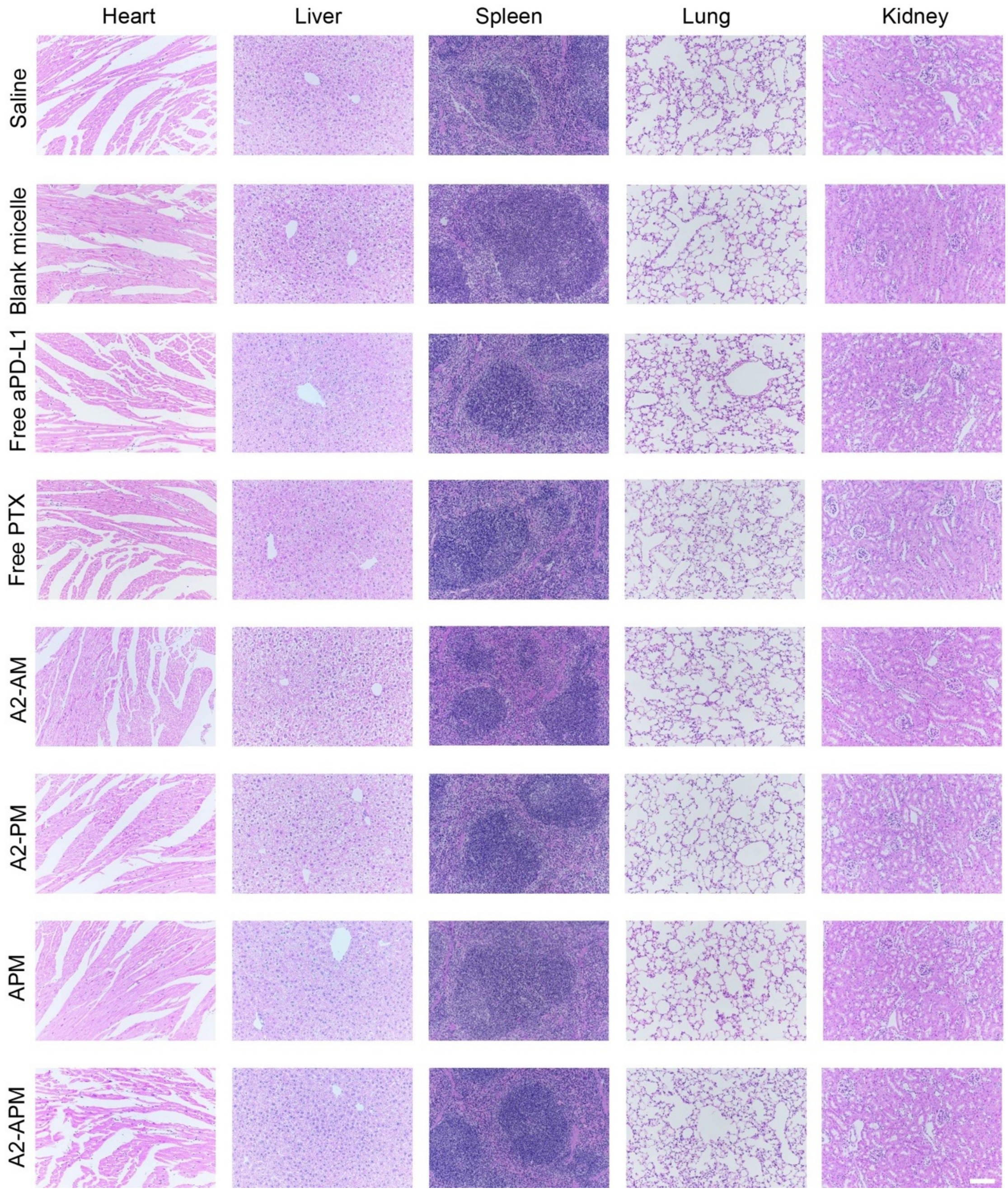

Supplementary Figure 27. H&E images of different organs after various treatments. H&E staining of the main tissues (heart, liver, spleen, lung, kidneys) of healthy mice receiving different treatments on 30 d after injection of various formulations. Scale bar = 100 μm. n = 3 biologically independent samples.

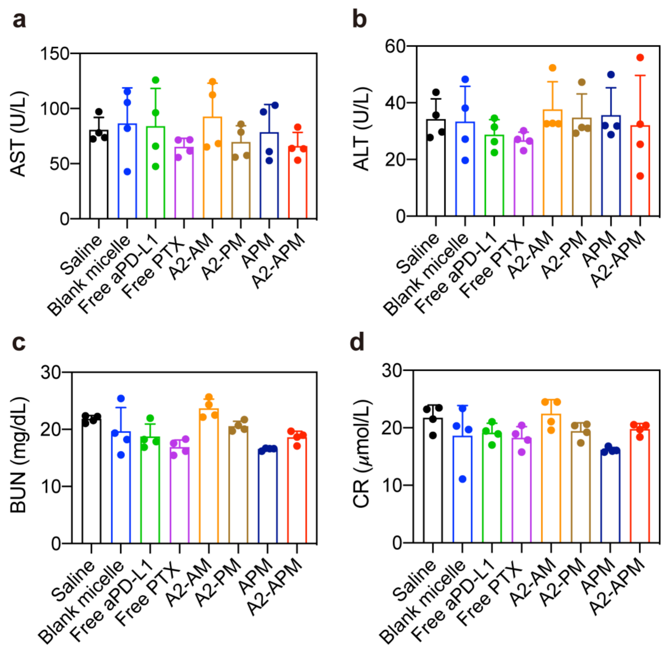

Supplementary Figure 28. Hematological indicators of the biosafety of various treatments in healthy mice. Hematological indexes of healthy mice at 30 d after injection of different treatments. **a** AST levels after treatments. **b** ALT levels after treatments. **c.** BUN levels after treatments. **d** Creatinine levels after treatments. n = 4 biologically independent samples, data are presented as mean ± SD.

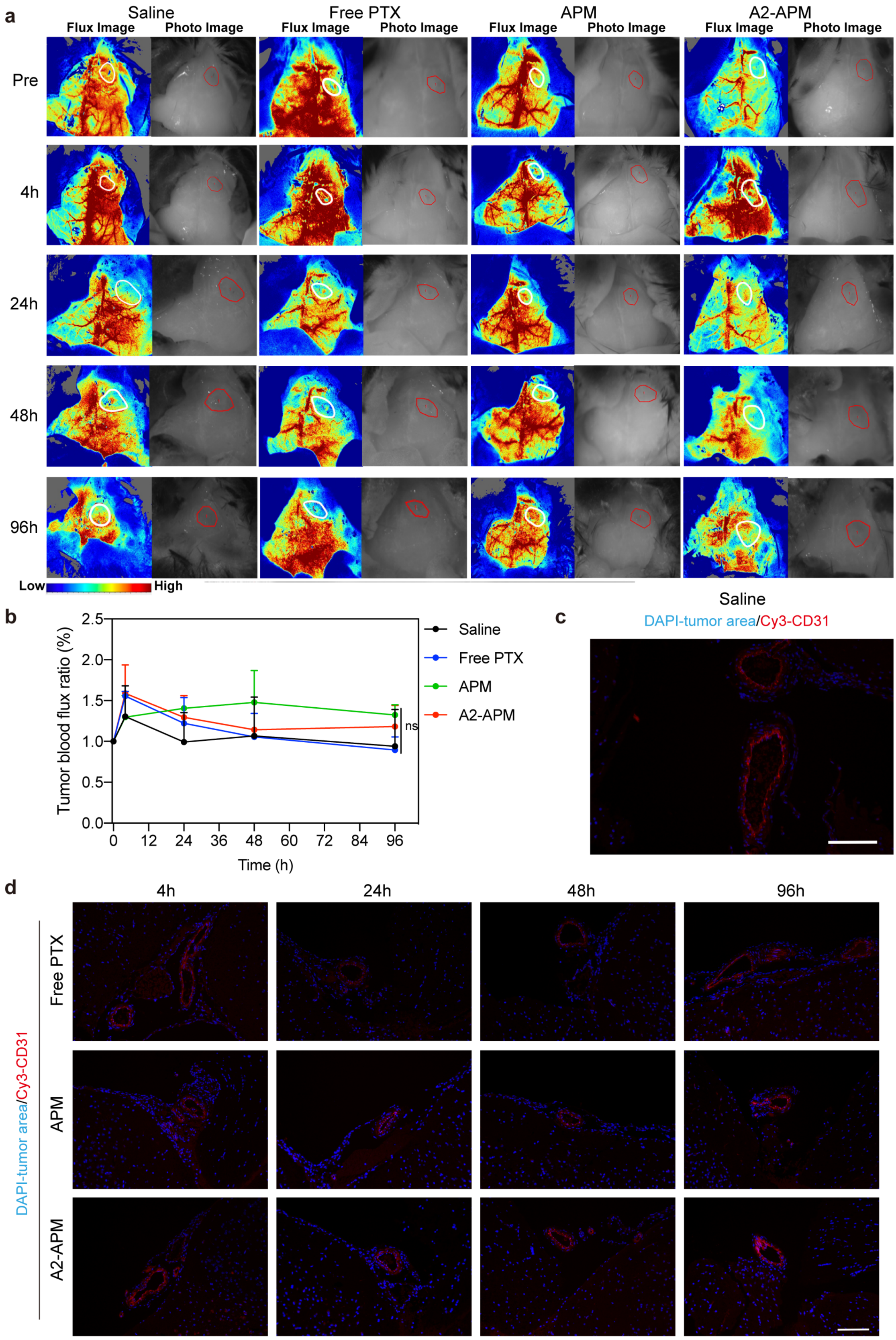

Supplementary Figure 29. Biosafety of different treatments by cerebral vessels images of GBM tumor-bearing mice. a, b Flux imaging and signal intensities ratio

at tumor sites at 0, 4, 24, 48 and 96 h after i.v. injection of saline, free PTX, APM and A2-APM into GL261 tumor bearing-mice (n = 3). The white lines in flux image and red lines in photo image indicate the tumor area. **c, d** Representative immunofluorescent images of cerebral vessels at 4, 24, 48 and 96 h after i.v. injection of GL261 tumor mice with saline, free PTX, APM and A2-APM (n = 3; Blue: DAPI; Red: Cy3-CD31; scale bar = 100μm). Statistical significance was calculated by one-way ANOVA with Fisher's LSD test.

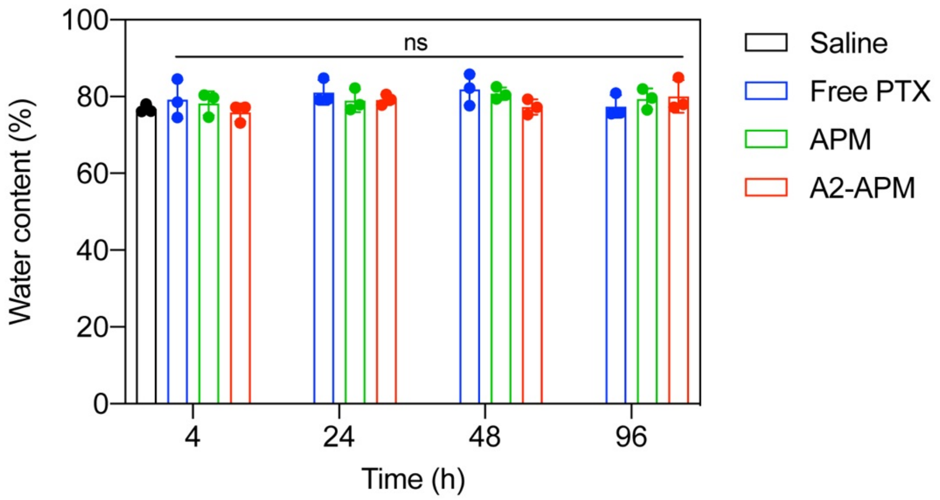

Supplementary Figure 30. Brain water content of GBM tumor-bearing mice after different treatments. Brain water content at 4, 24, 48 and 96 h after i.v. injection of GL261 tumor mice with saline, free PTX, APM and A2-APM. n = 3. Statistical significance was calculated by one-way ANOVA with Fisher's LSD test.

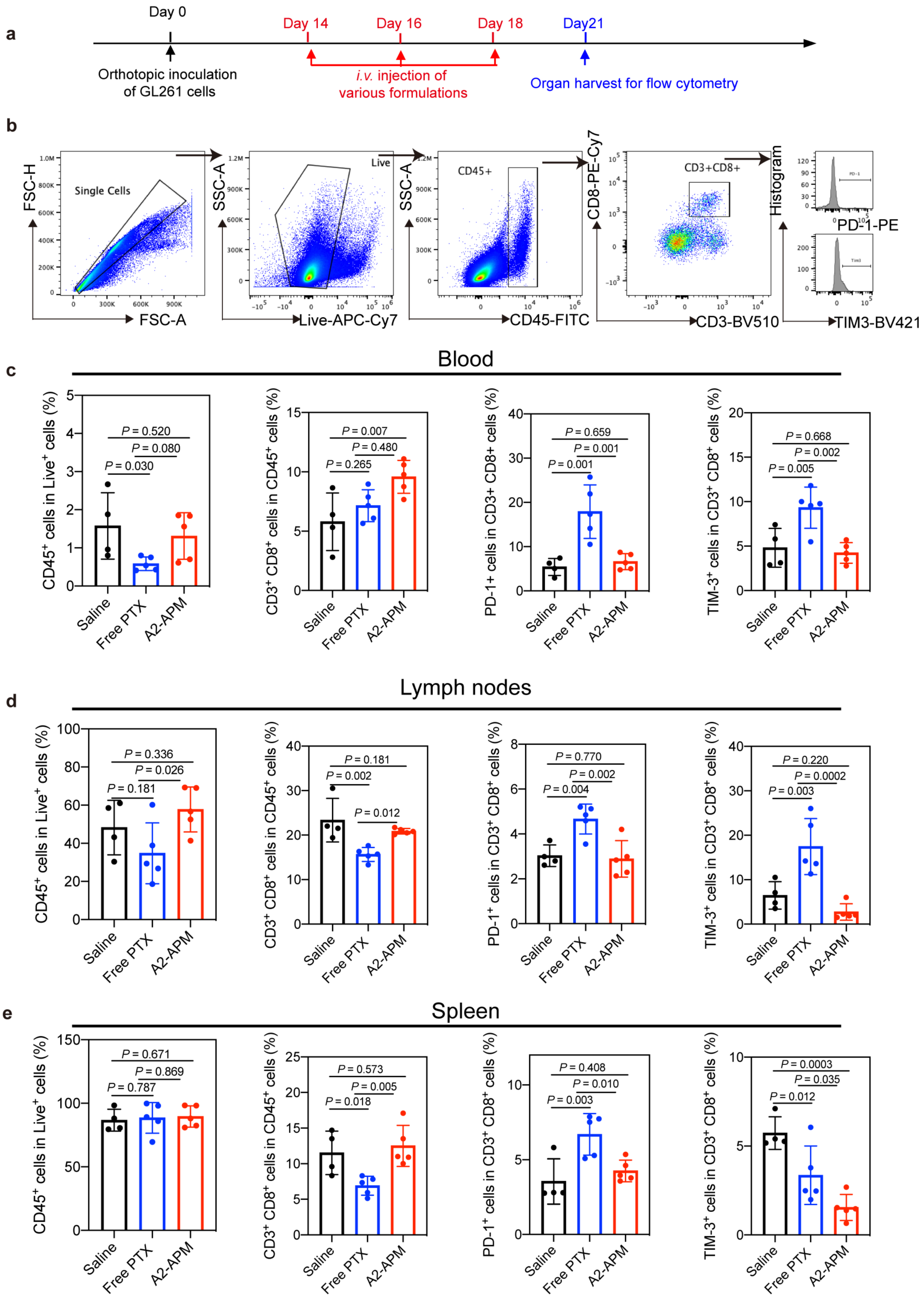

Supplementary Figure 31. A2-APM can effectively mitigate lymphodepletion. a Schematic of the treatment regimen. **b** Gating strategy to sort $CD45^+$ cells, $CD3^+$ $CD8^+$ T cells, $PD\text{-}1^+$ cells, and $TIM\text{-}3^+$ cells in tumor tissues. **c-e** Quantification of blood, lymph nodes and spleen-infiltrating $CD45^+$ cells, $CD3^+$ $CD8^+$ T cells in $CD45^+$

cells and PD-1$^+$, TIM-3$^+$ cells in CD3$^+$ CD8$^+$ T cells three days after treatment with saline, free PTX and A2-APM (PTX conc. = 5mg/kg). n =4 saline treated mice and 5 free PTX and A2-APM treated mice. Statistical significance was calculated by one-way ANOVA with Fisher's LSD test.

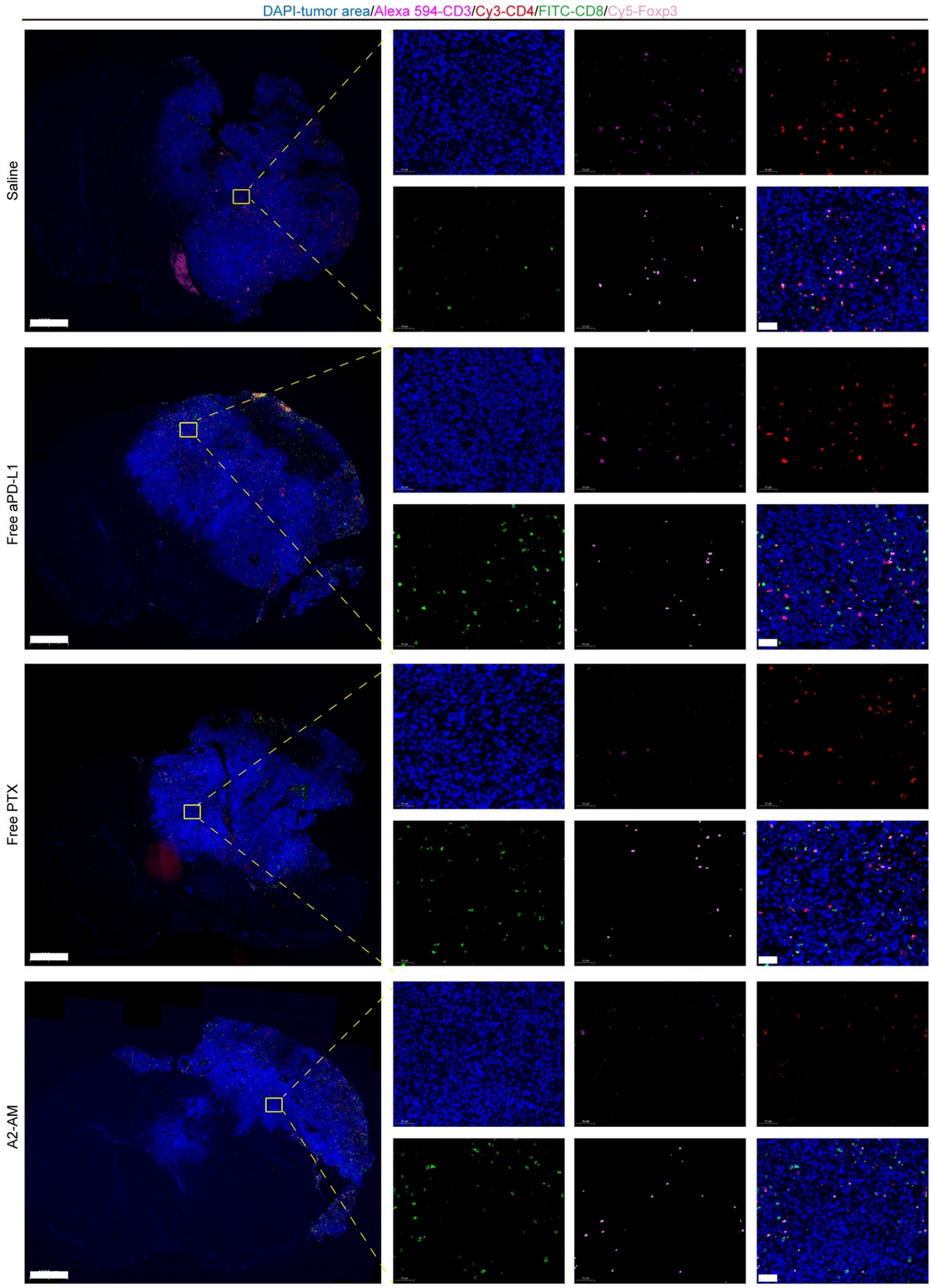
DAPI-tumor area/Alexa 594-CD3/Cy3-CD4/FITC-CD8/Cy5-Foxp3
Saline
Free aPD-L1
Free PTX
A2-AM

(Continued from previous page)

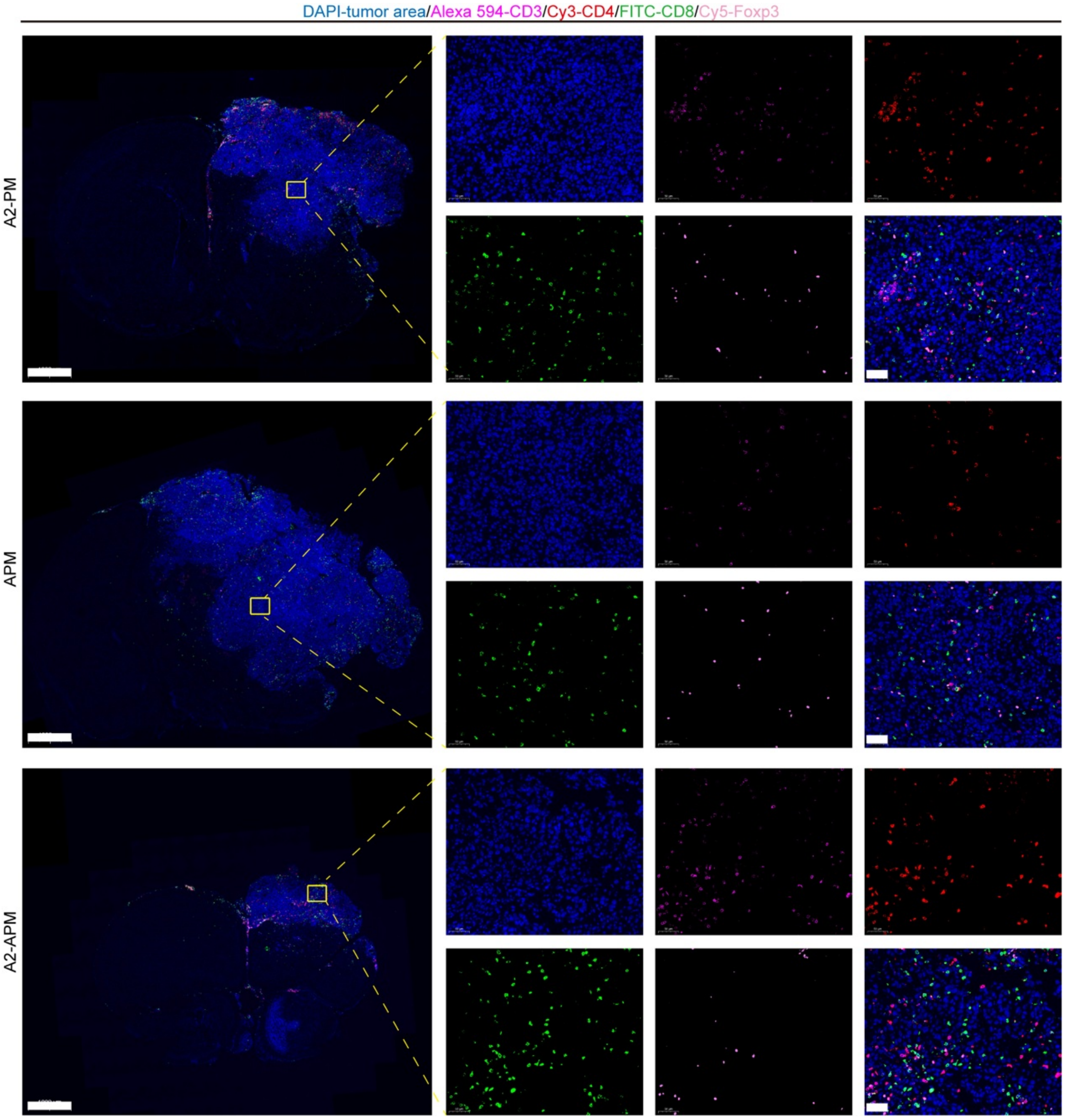

Supplementary Figure 32. A2-APM stimulates intratumor T cell proliferation and infiltration. Representative T cell immunofluorescence of tumor tissue sections three days after two injections of different formulas. Blue: DAPI; green: FITC-CD8; rose red: Alexa 594-CD4; red: Cy3-CD3; pink: Cy5-Foxp3. Scale bar of the left panel = 1000 μm, scale bar of the right panel = 50 μm. n = 3 biologically independent samples.

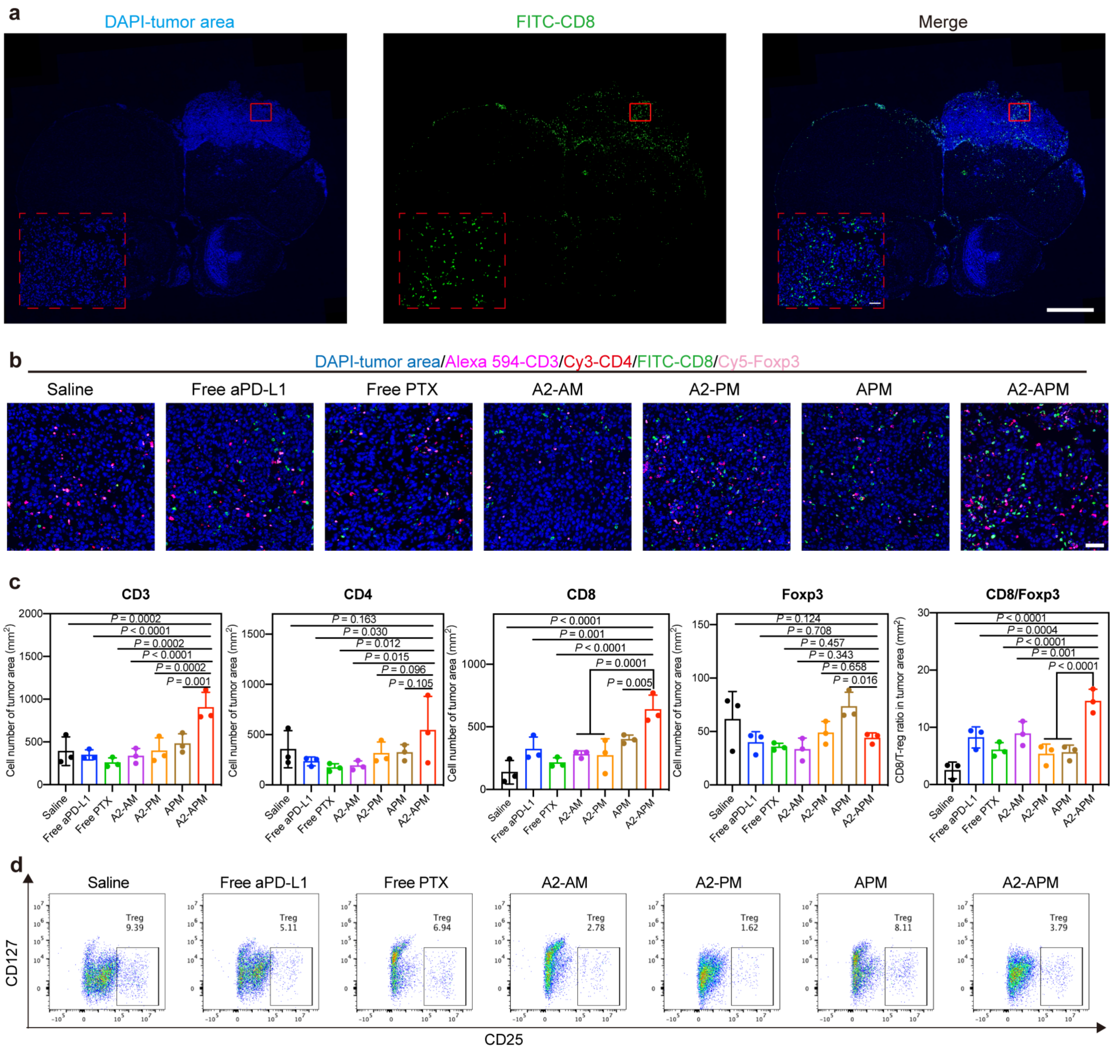

Supplementary Figure 33. A2-APM promotes CD8 T cell infiltration and proliferation and decreases Treg ratio in GL261 tumor mice. a Representative immunofluorescent image of tumor-infiltrating CD8$^+$ T cells three days after treatment with A2-APM (blue: DAPI; green: FITC-CD8; scale bar = 1000μm). Insets: Image after 200× magnification of the right red frame, scale bar = 50 μm. **b**, **c** Representative immunofluorescent images and quantification of tumor-infiltrating T cells three days after two treatments with saline, free aPD-L1, free PTX, A2-AM, A2-PM, APM and A2-APM (blue: DAPI; green: FITC-CD8; rose red: Alexa 594-CD4; red: Cy3-CD3; pink: Cy5-Foxp3; scale bar = 50 μm). **d** Representative flow cytometry dot plots of tumor-infiltrating CD25^{hi}CD127^{lo} T cells three days after two treatments with saline, free aPD-L1, free PTX, A2-AM, A2-PM, APM and A2-APM Insets: the subsets of CD25^{hi}CD127^{lo} T cells in CD45$^+$CD4$^+$ cells; the

numbers indicate the percentage of $CD25^{hi}CD127^{lo}$ T cells in $CD45^{+}CD4^{+}$ cells after various treatments. n = 3 biologically independent samples. Statistical significance was calculated by one-way ANOVA with Fisher's LSD test.

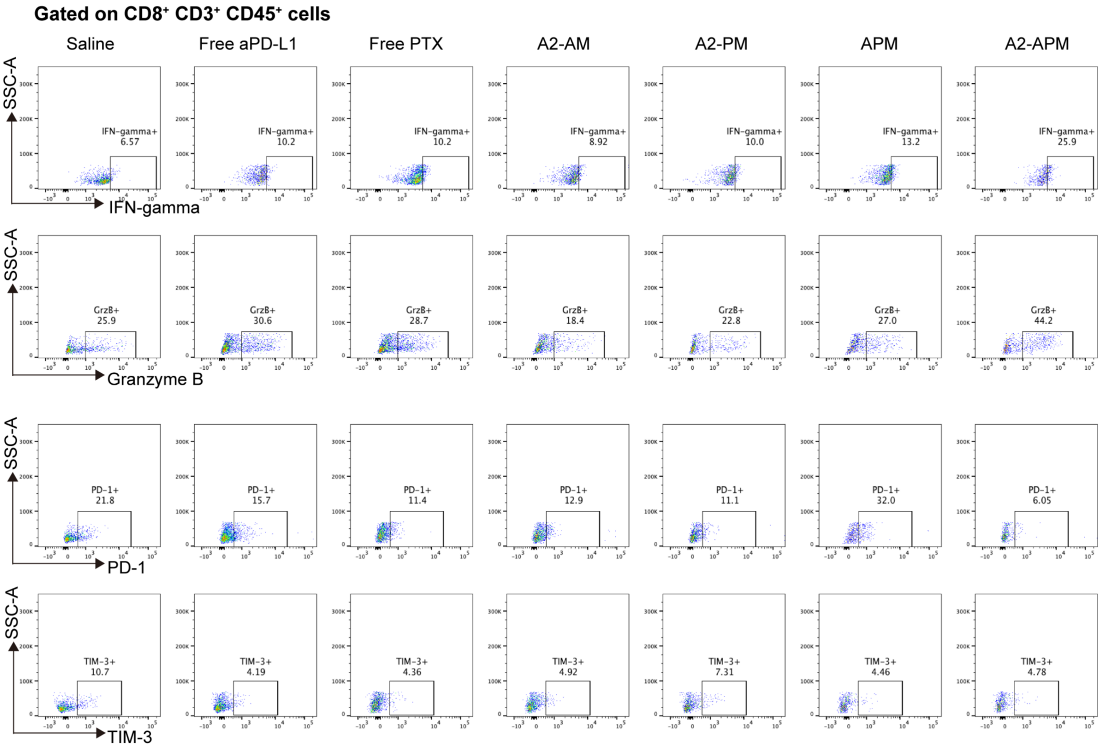

Supplementary Figure 34. A2-APM treatment increases the proportion of cytotoxic $CD8^{+}$ T cells and decreases the proportion of exhausted $CD8^{+}$ T cells in GL261 tumor mice. Representative flow cytometric contour plots of tumor-infiltrating IFNγ^{+}, Granzyme B^{+} ($GrzB^{+}$), PD-1^{+} and TIM-3^{+} T cells three days after two treatments with saline, free aPD-L1, free PTX, A2-AM, A2-PM, APM and A2-APM. Insets: the subsets of IFNγ^{+}, Granzyme B^{+} ($GrzB^{+}$), PD-1^{+} and TIM-3^{+} T cells in $CD3^{+}CD8^{+}$ T cells; the numbers indicate the percentage of IFNγ^{+}, Granzyme B^{+} ($GrzB^{+}$), PD-1^{+} and TIM-3^{+} T cells in $CD3^{+}CD8^{+}$ T cells after various treatments. Saline treated mice (n = 3), free aPD-L1, free PTX, A2-AM, A2-PM, APM and A2-APM treated mice (n = 4).

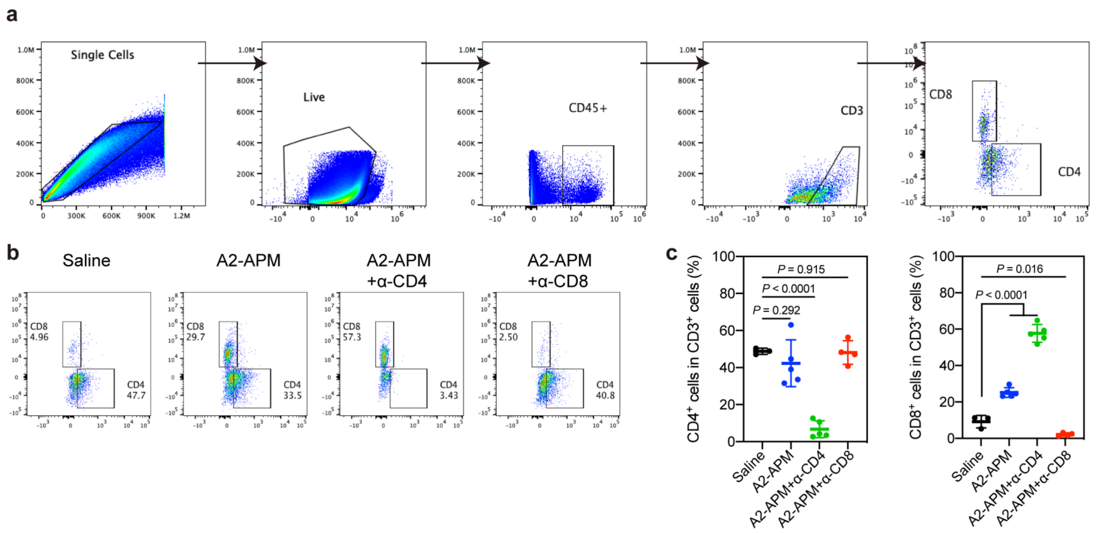

Supplementary Figure 35. Depletion of CD8^{+} T cells abrogates A2-APM treatment efficacy. a Gating strategy for CD4^{+} and CD8^{+} T cells. **b, c** Depletion was confirmed on day 29 in the TME by flow cytometry. All data are presented as individual values and the mean ± SEM. Statistical significance was calculated by one-way ANOVA with Fisher's LSD test. Saline treated mice (n = 3), A2-APM (n = 5) treated mice, A2-APM + α-CD4 treated mice (n = 5), A2-APM + α-CD8 treated mice (n = 4).

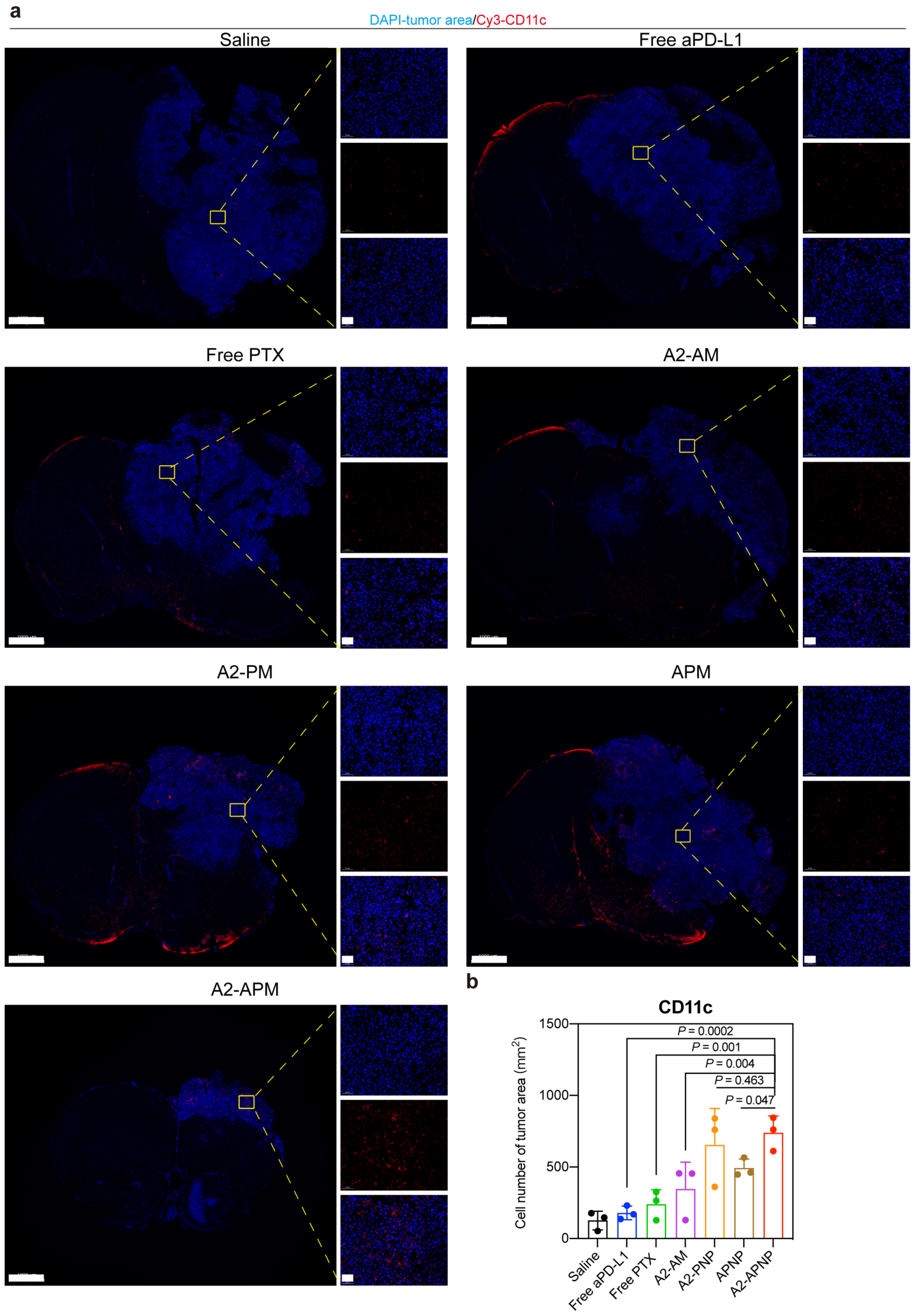

Supplementary Figure 36. A2-APM stimulates intratumor DC proliferation. a, b. Representative immunofluorescence images and their qualification of DC in tumor tissue sections three days after two injections of different treatments. Blue: DAPI; red:

Cy3-CD11c. Scale bar of the left panel = 1000 μm, scale bar of the right panel = 50 μm. Data are presented as means ± SD, n = 3 biologically independent samples. Statistical significance was calculated by one-way ANOVA with Fisher's LSD test.

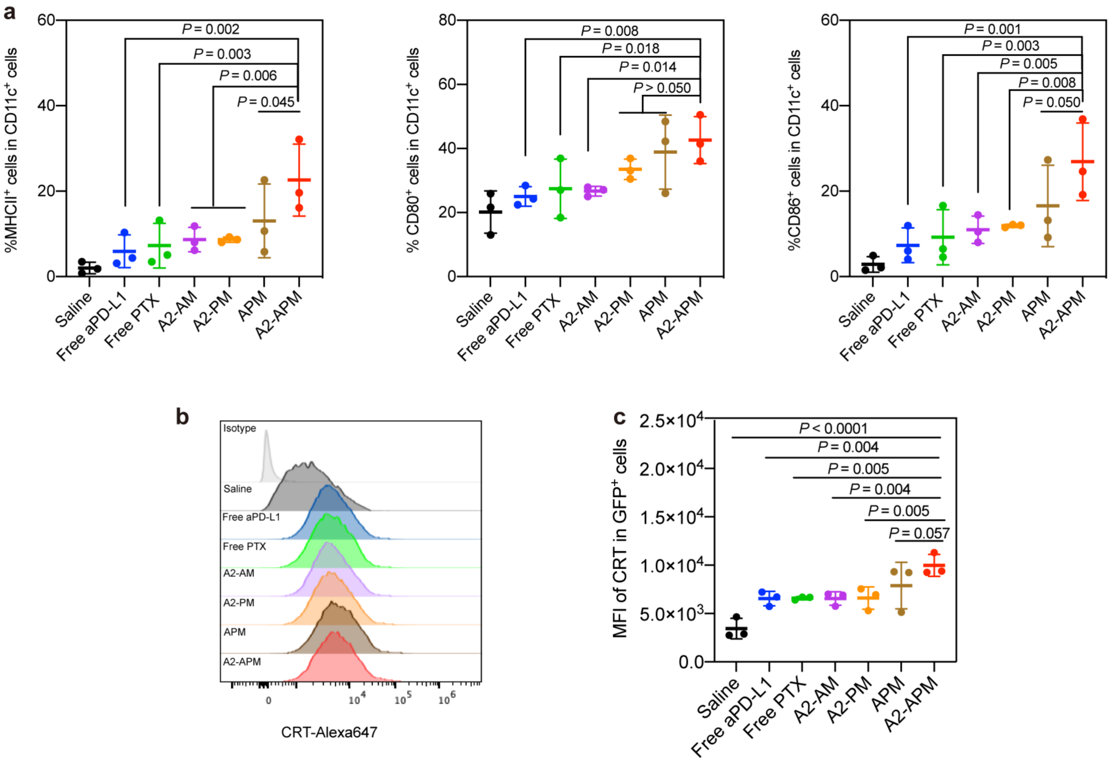

Supplementary Figure 37. A2-APM treatment promotes DC maturation by ICD effect in GL261 tumor mice. a The percentages of each marker's expression were calculated based on Figure 6a. **b** Representative histograms display CRT expression levels compared to isotype controls. **c** MFI of each marker's expression were calculated based on (b). All statistics are expressed as mean ± SD, n = 3. Statistical significance was calculated by one-way ANOVA with Fisher's LSD test.

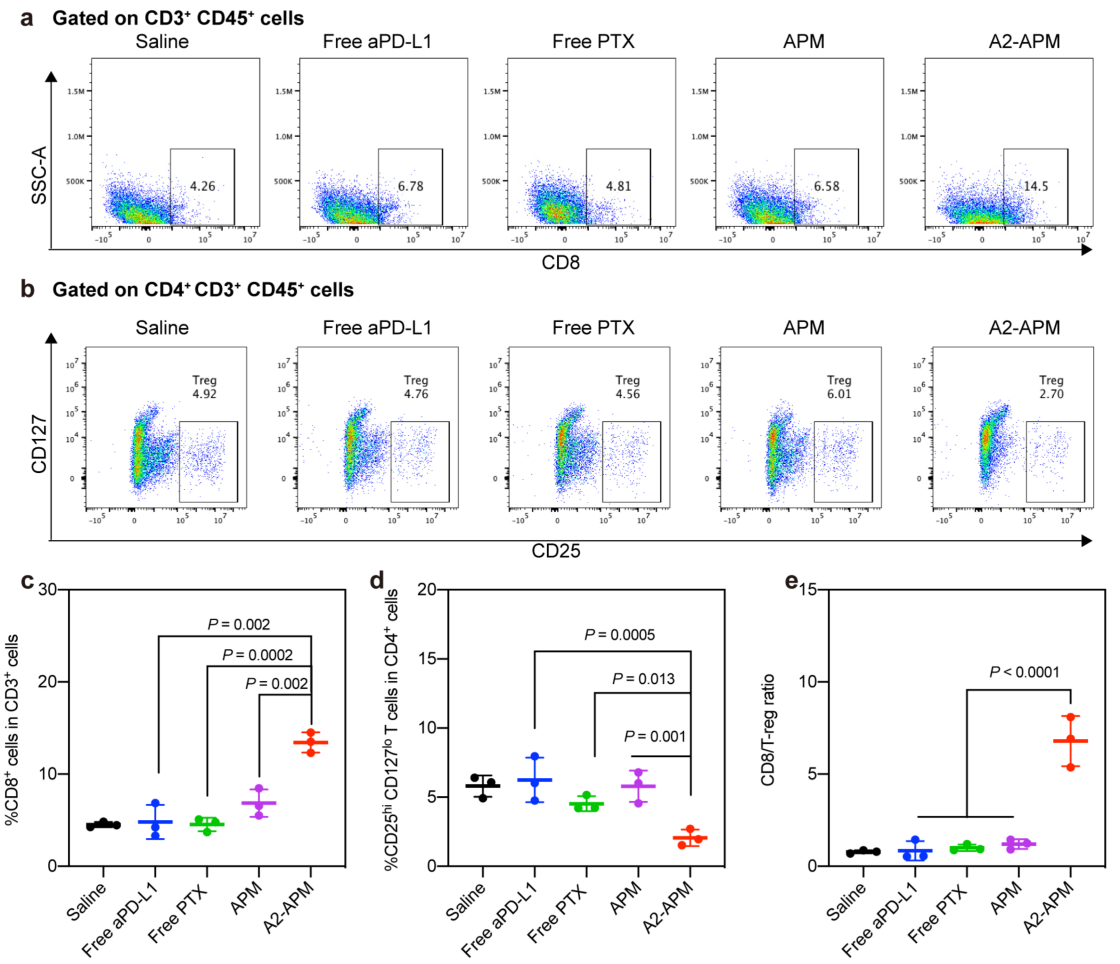

Supplementary Figure 38. A2-APM promotes CD8 T cell infiltration and proliferation and decreases Treg ratio in G422 tumor mice. **a** Representative flow cytometric contour plots of tumor-infiltrating CD8^{+} T cell three days after two treatments with saline, free aPD-L1, free PTX, APM and A2-APM. Insets: the subsets of CD8^{+} T cells in CD45^{+}CD3^{+} cells; the numbers indicate the percentage of CD8^{+} T cells in CD45^{+}CD3^{+} cells after various treatments. **b.** Representative flow cytometric dot plots of tumor-infiltrating CD25^{hi}CD127^{lo} Treg cells three days after two treatments with saline, free aPD-L1, free PTX, APM and A2-APM. **c-e.** Quantification of tumor-infiltrating T cells three days after two treatments with saline, free aPD-L1, free PTX, APM and A2-APM. All statistics are expressed as mean ± SD, n = 3. Statistical significance was calculated by one-way ANOVA with Fisher's LSD test.

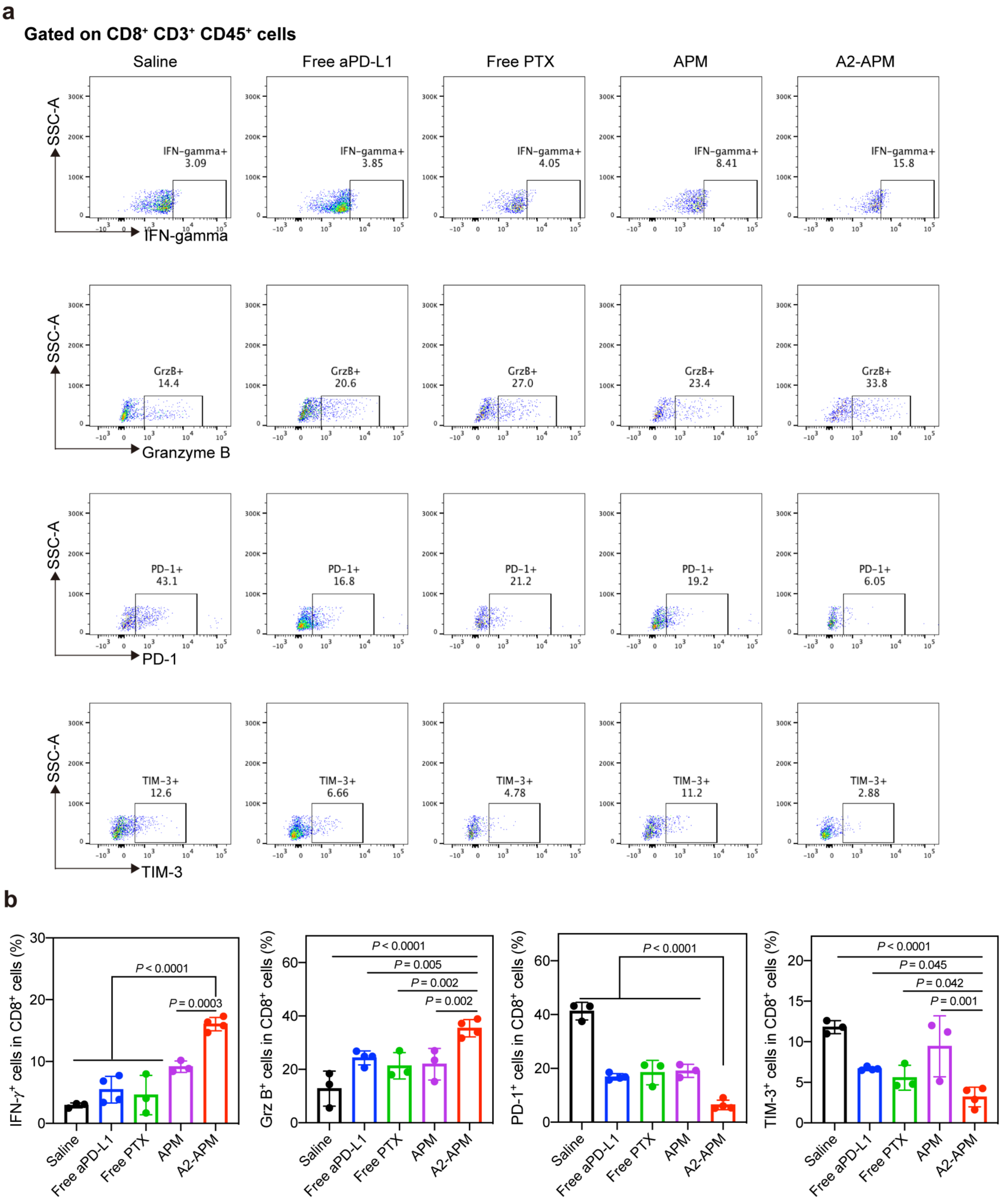

Supplementary Figure 39. A2-APM treatment increases the proportion of cytotoxic CD8 T cells and decreases the proportion of exhausted CD8 T cells in G422 tumor mice. a Representative flow cytometric contour plots of tumor-infiltrating IFNγ$^+$, Granzyme B$^+$ (GrzB$^+$), PD-1$^+$and TIM-3$^+$ T cells three days after two treatments with saline, free aPD-L1, free PTX, A2-AM, A2-PM, APM and A2-APM. Insets: the subsets of IFNγ$^+$, Granzyme B$^+$ (GrzB$^+$), PD-1$^+$and TIM-3$^+$ T cells in CD3$^+$CD8$^+$ T cells; the numbers indicate the percentage of IFNγ$^+$, Granzyme B$^+$ (GrzB$^+$), PD-1$^+$and TIM-3$^+$ T cells in CD3$^+$CD8$^+$ T cells after various treatments.

b Quantification of tumor-infiltrating IFNγ^{+}, Granzyme B^{+} (GrzB^{+}), PD-1^{+}and TIM-3^{+} T cells three days after two treatments with saline, free aPD-L1, free PTX, A2-AM, A2-PM, APM and A2-APM. All statistics are expressed as mean ± SD, n = 3. Statistical significance was calculated by one-way ANOVA with Fisher's LSD test.

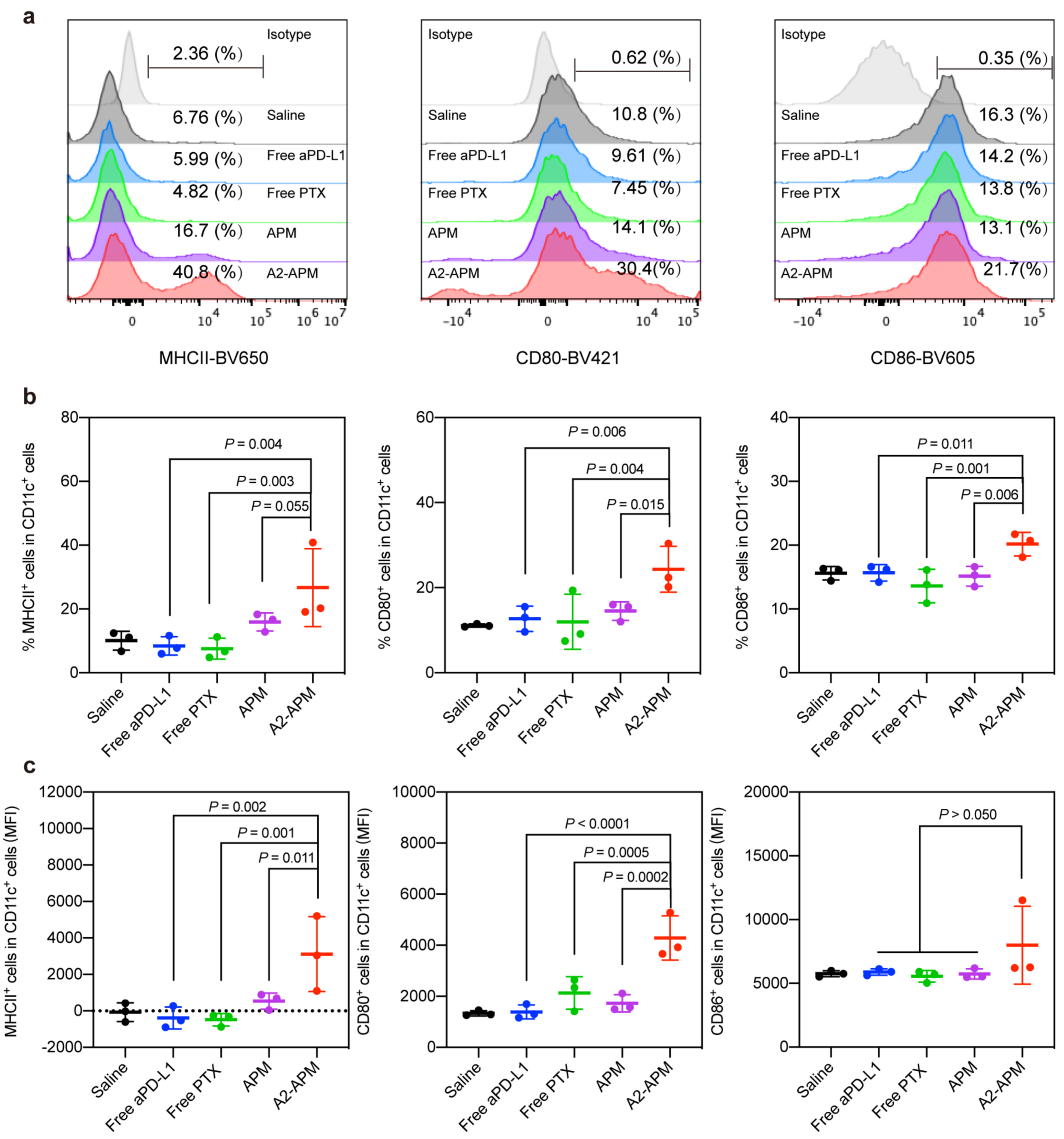

Supplementary Figure 40. A2-APM treatment promotes DC maturation in G422 tumor mice. Representative flow cytometry histogram of tumor-infiltrating CD11c^{+} cells three days after two treatments with saline, free aPD-L1, free PTX, A2-AM, A2-PM, APM and A2-APM. Levels of MHCII, CD80 and CD86 were evaluated *via* flow cytometry. **a** Representative histograms display each marker's expression levels

compared to isotype controls. The percentages **b** and MFI **c** of each marker's expression were calculated based on **a.** All statistics are expressed as mean ± SD, n = 3. Statistical significance was calculated by one-way ANOVA with Fisher's LSD test.

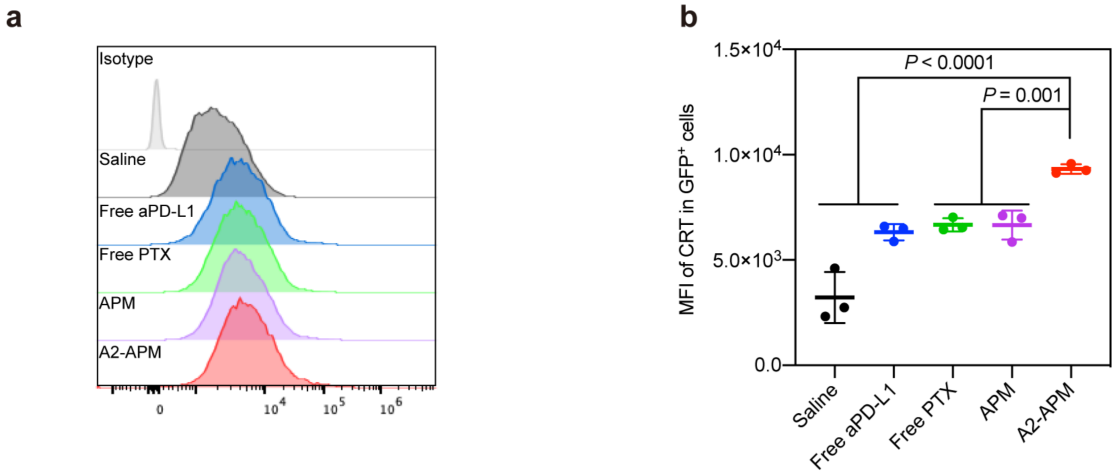

Supplementary Figure 41. Analysis of the exposure of CRT on G422 tumor cells after various treatments. Representative flow cytometry histogram **a** and quantification **b** of the exposure of CRT on tumor cells three days after two treatments with saline, free aPD-L1, free PTX, APM and A2-APM. n = 3 biologically independent samples. Statistical significance was calculated by one-way ANOVA with Fisher's LSD test.

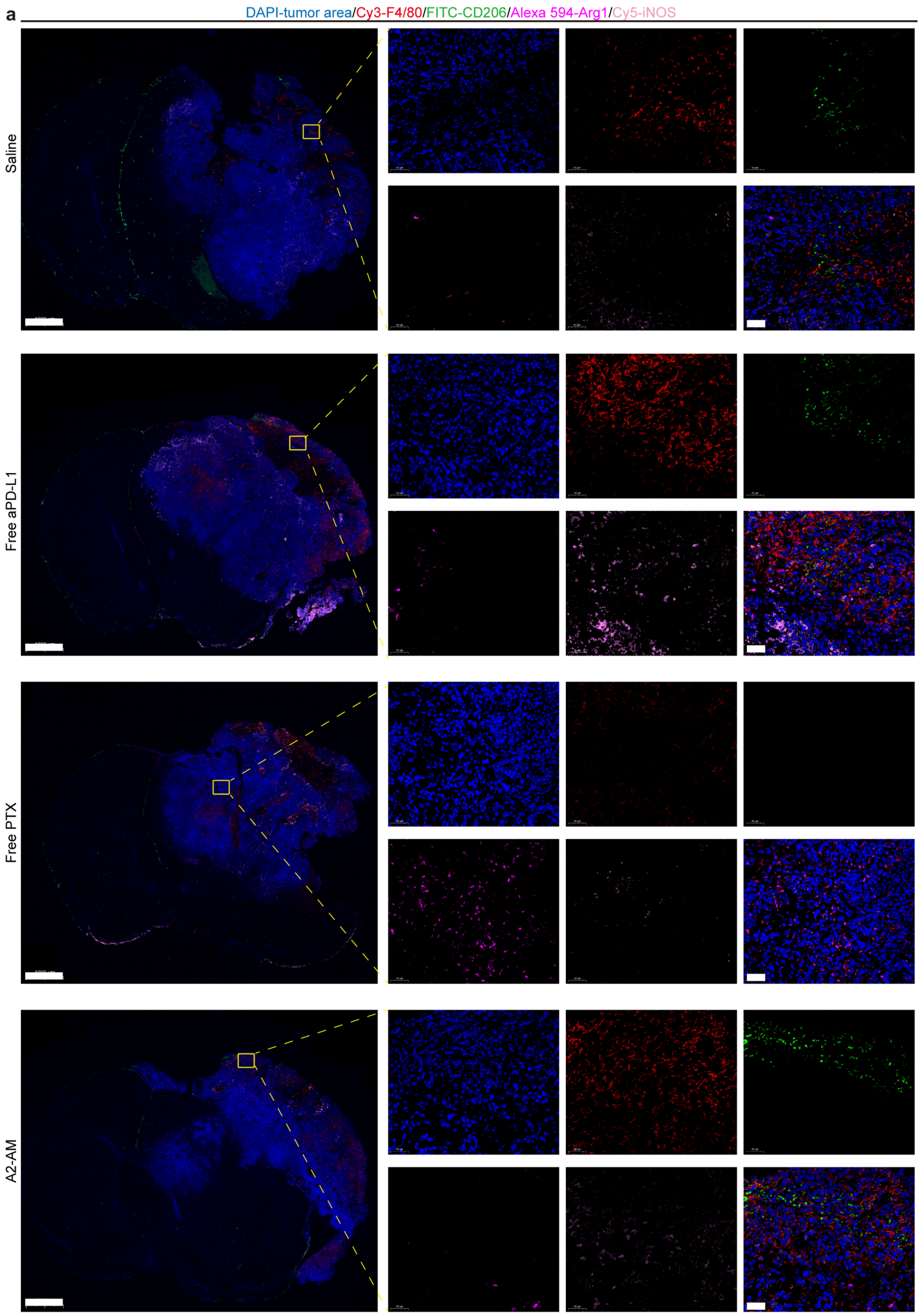
a
DAPI-tumor area/Cy3-F4/80/FITC-CD206/Alexa 594-Arg1/Cy5-iNOS
Saline
Free aPD-L1
Free PTX
A2-AM

(Continued from previous page)

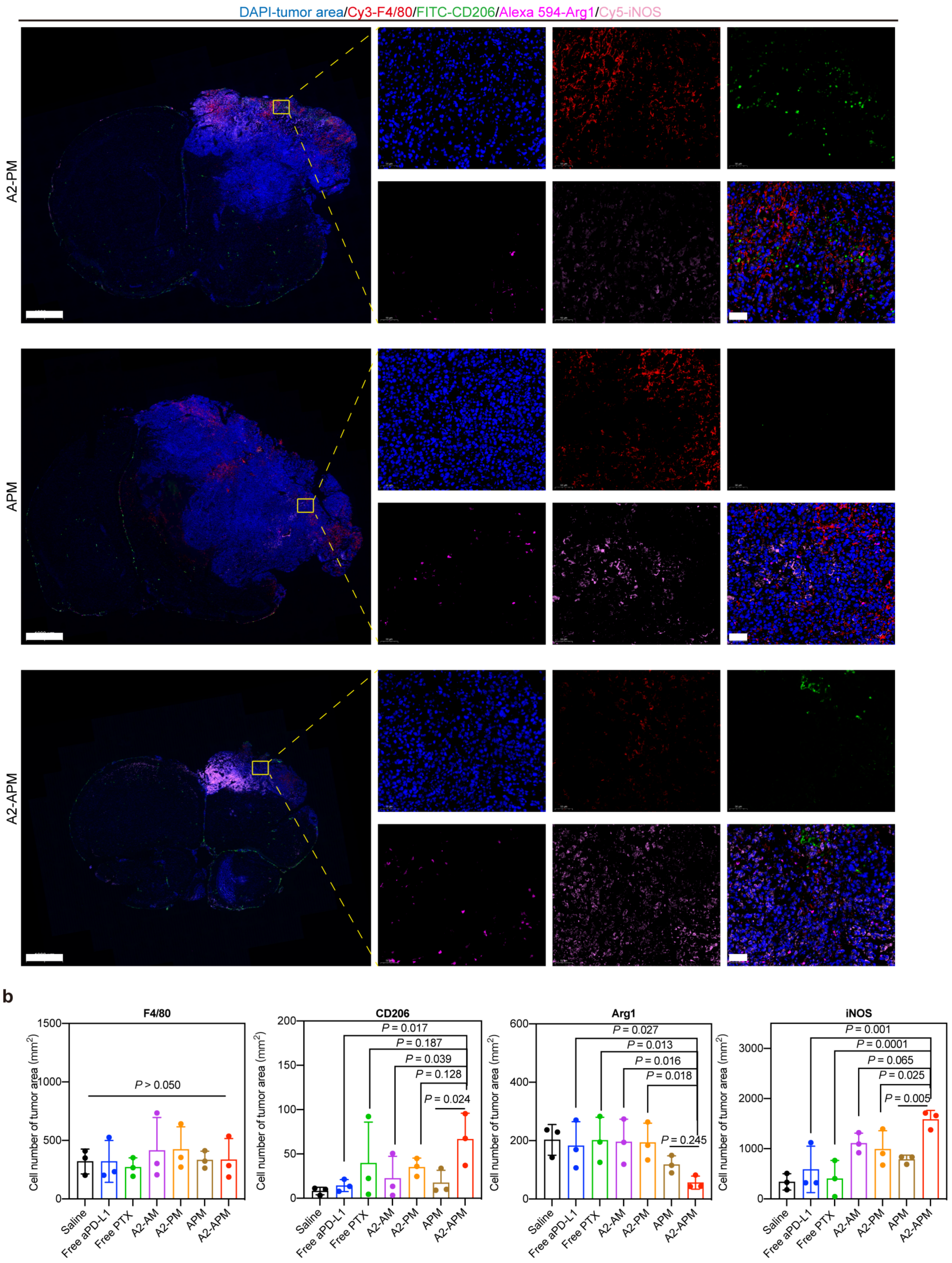

Supplementary Figure 42. A2-APM alteres macrophage polarization in tumor tissues. a, b. Representative immunofluorescence images and their qualification of macrophage in tumor tissue sections three days after two injections of different treatments. Blue: DAPI; green: FITC-CD206; rose red: Alexa 594-Arg1; red: Cy3-F4/80; pink: Cy5-iNOS. Scale bar of the left panel = 1000 μm, scale bar of the

right panel = 50 μm. Data are presented as means ± SD, n = 3 biologically independent samples. Statistical significance was calculated by one-way ANOVA with Fisher's LSD test.

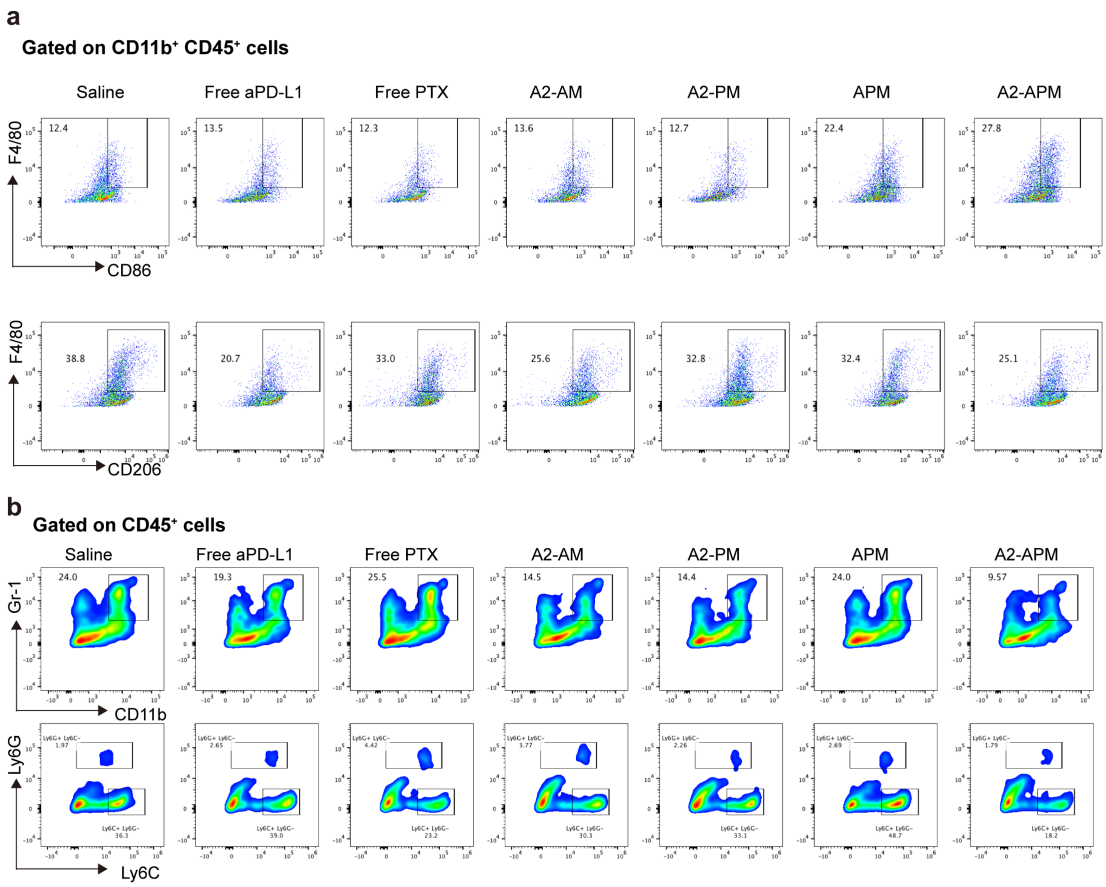

Supplementary Figure 43. A2-APM treatment results in pro-inflammatory transformation of macrophages and decreases myeloid-derived suppressor cells in GL261 tumor mice. a Representative flow cytometric contour plots of tumor-infiltrating CD86$^+$F4/80$^+$ and CD206$^+$F4/80$^+$ cells three days after two treatments with saline, free aPD-L1, free PTX, A2-AM, A2-PM, APM and A2-APM. Insets: the subsets of CD86$^+$F4/80$^+$ cells in CD45$^+$CD11b$^+$ cells; the numbers indicate the percentage of CD86$^+$F4/80$^+$ and CD206$^+$F4/80$^+$ cells in CD11b$^+$CD45$^+$ cells after various treatments. **b** Representative flow cytometric contour plots of tumor-infiltrating Gr-1$^+$CD11b$^+$, Ly6C$^+$ and Ly6G$^+$ cells three days after two treatments with saline, free aPD-L1, free PTX, A2-AM, A2-PM, APM and A2-APM. Insets: the subsets of Gr-1$^+$ CD11b$^+$ in CD45$^+$ cells, Ly6C$^+$ and Ly6G$^+$ cells in CD11b$^+$CD45$^+$ cells; the numbers indicate the percentage of r-1$^+$CD11b$^+$ in CD45$^+$ cells, Ly6C$^+$ and Ly6G$^+$ cells in CD11b$^+$CD45$^+$ cells after various treatments. n = 3 saline treated mice, n = 4 free aPD-L1, free PTX, A2-AM, A2-PM, APM and

A2-APM treated mice.

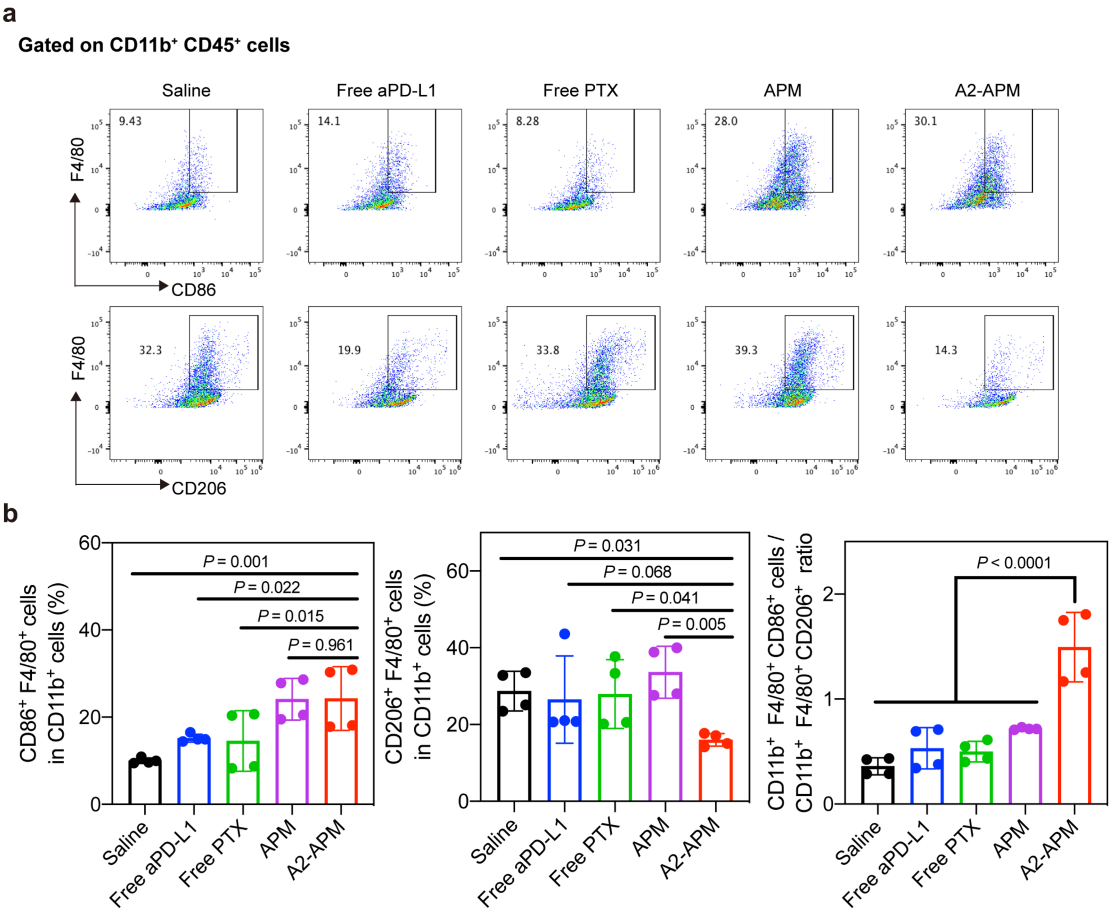

Supplementary Figure 44. A2-APM treatment results in pro-inflammatory transformation of macrophages in G422 tumor mice. a Representative flow cytometric contour plots of tumor-infiltrating CD86$^+$F4/80$^+$ and CD206$^+$F4/80$^+$ cells three days after two treatments with saline, free aPD-L1, free PTX, APM and A2-APM. Insets: the subsets of CD86$^+$F4/80$^+$ and CD206$^+$F4/80$^+$ cells in CD11b$^+$CD45$^+$ cells; the numbers indicate the percentage of CD86$^+$F4/80$^+$ and CD206$^+$F4/80$^+$ cells in CD11b$^+$CD45$^+$ cells after various treatments. **b** Quantification of tumor-infiltrating CD86$^+$F4/80$^+$ and CD206$^+$F4/80$^+$ cells three days after two treatments with saline, free aPD-L1, free PTX, APM and A2-APM. All statistics are expressed as mean ± SD, n = 4. Statistical significance was calculated by one-way ANOVA with Fisher's LSD test.

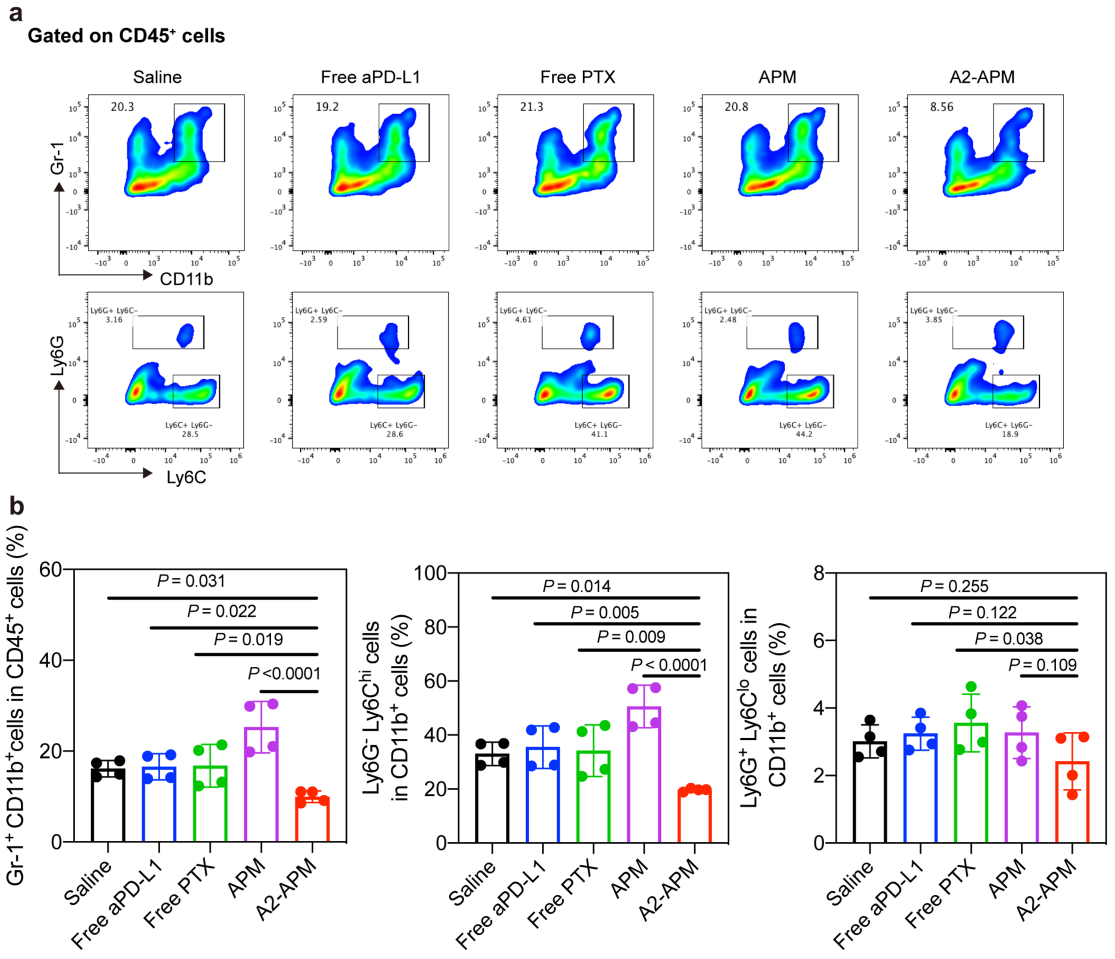

Supplementary Figure 45. A2-APM treatment decreases myeloid-derived suppressor cells in G422 tumor mice. a Representative flow cytometric contour plots of tumor-infiltrating Gr-1$^+$CD11b$^+$, Ly6C$^+$ and Ly6G$^+$ cells three days after two treatments with saline, free aPD-L1, free PTX, APM and A2-APM. Insets: the subsets of Gr-1$^+$CD11b$^+$ in CD45$^+$ cells, Ly6C$^+$ and Ly6G$^+$ cells in CD11b$^+$CD45$^+$ cells; the numbers indicate the percentage of r-1$^+$CD11b$^+$ in CD45$^+$ cells, Ly6C$^+$ and Ly6G$^+$ cells in CD11b$^+$CD45$^+$ cells after various treatments. **b** Quantification of tumor-infiltrating Gr-1$^+$CD11b$^+$, Ly6C$^+$ and Ly6G$^+$ cells three days after two treatments with saline, free aPD-L1, free PTX, APM and A2-APM. All statistics are expressed as mean ± SD, n = 4. Statistical significance was calculated by one-way ANOVA with Fisher's LSD test.

All samples were sorted with anti-CD45 magnetic beads

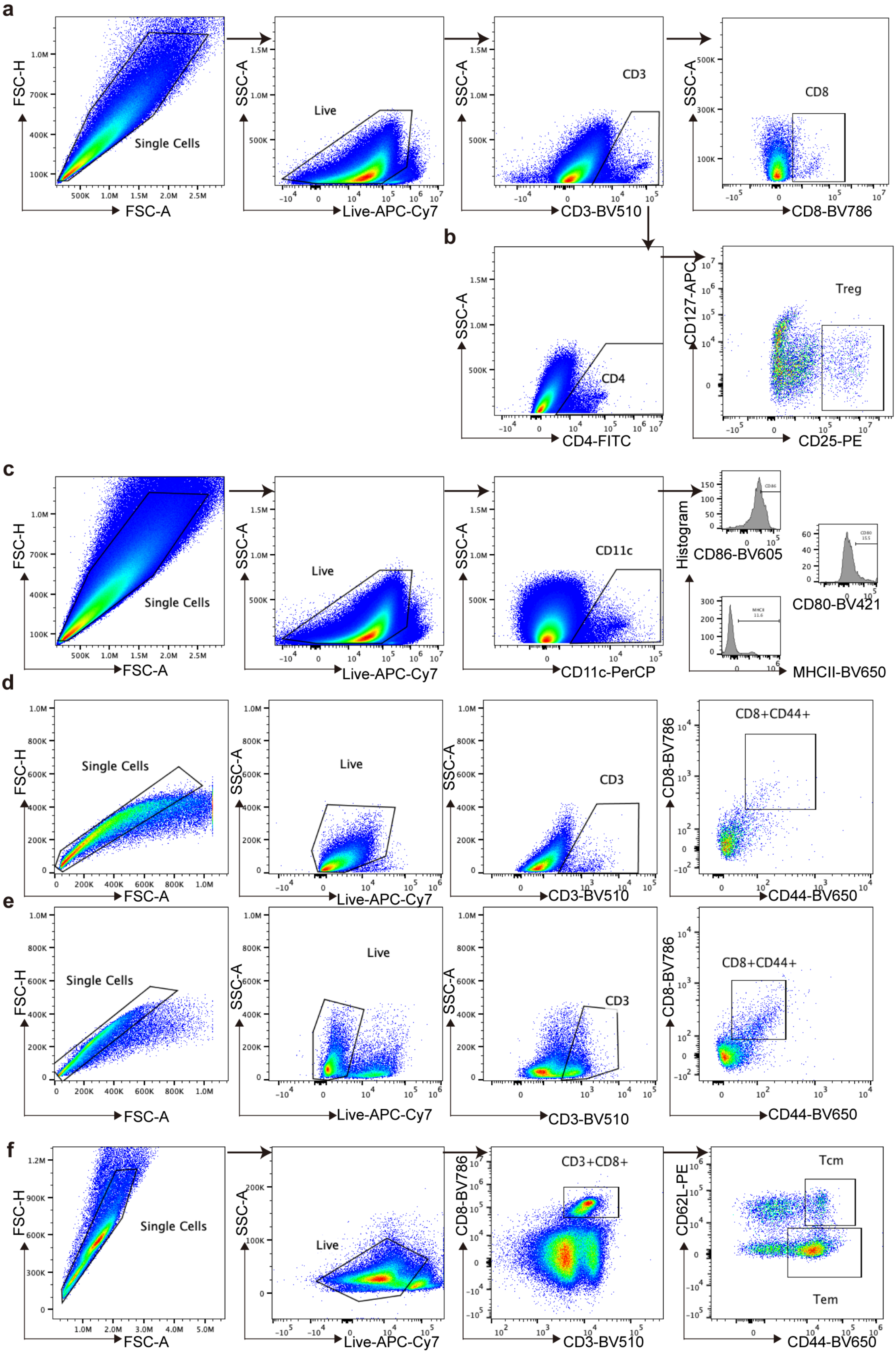

Supplementary Figure 46. Graphical account for flow cytometry gating

strategies of CD8$^+$ T cells, Tregs, DCs and T memory cells. a Gating strategy to sort CD8$^+$ T cells in tumor tissues gating on CD45 Live$^+$ CD3$^+$ cells presented on Figure 5a and Supplementary Figure 38a. **b** Gating strategy to sort CD25^{hi} CD127^{lo} Tregs in tumor tissues gating on CD45$^+$Live$^+$CD4$^+$ cells presented on Supplementary Figure 33d and Supplementary Figure 38b. **c** Gating strategy to access the level of MHCII, CD80, and CD86 in tumor tissues gating on CD45$^+$Live$^+$CD11c$^+$ cells presented on Figure 2h, Supplementary Figure 37a and Supplementary Figure 40. **d, e** Gating strategy to sort CD44^{hi}CD8$^+$ memory T cells in (d) brain and (e) dLNs gating on CD45$^+$Live$^+$CD3$^+$ cells presented on Figure 7b. **f.** Gating strategy to sort CD44^{hi}CD62L^{lo} effector memory T cells in spleen tissues gating on CD45$^+$Live$^+$CD3$^+$CD8$^+$ cells presented on Figure 7d.

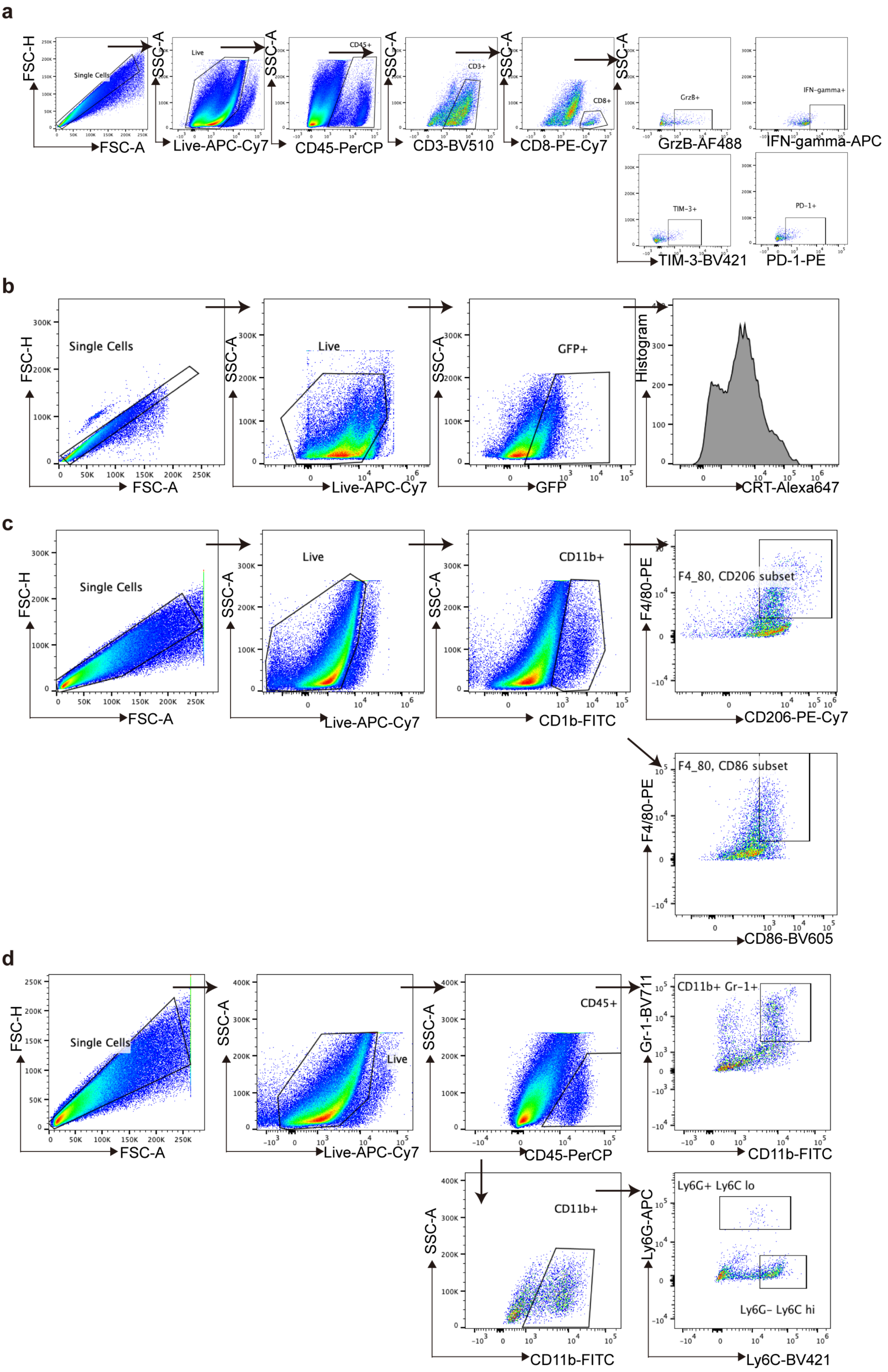

Supplementary Figure 47. Graphical account for flow cytometry gating strategies of CD8^{+} T cells, CRT, macrophages and MDSCs. a Gating strategy to

sort PD-1$^+$, TIM-3$^+$, GrzB$^+$ and IFN-γ$^+$ T cells in tumor tissues gating on CD3$^+$CD8$^+$ T cells, CD45$^+$Live$^+$CD3$^+$ cells presented on Supplementary Figure 34 and Supplementary Figure 39a. **b** Gating strategy to sort CRT$^+$ cells in tumor tissues gating on GFP$^+$Live$^+$ cells presented on Supplementary Figure 37b and Supplementary Figure 41a. **c** Gating strategy to sort F4/80$^+$CD206$^+$ and F4/80$^+$CD86$^+$ cells in tumor tissues gating on CD45$^+$Live$^+$CD11b$^+$ cells presented on Supplementary Figure 43a and 44a. **d** Gating strategy to sort Gr-1$^+$CD11b$^+$, Ly6C$^+$Ly6G$^-$ and Ly6C$^-$Ly6G$^+$ MDSCs gating on CD45$^+$Live$^+$ cells presented on Supplementary Figure 43b and 45a.

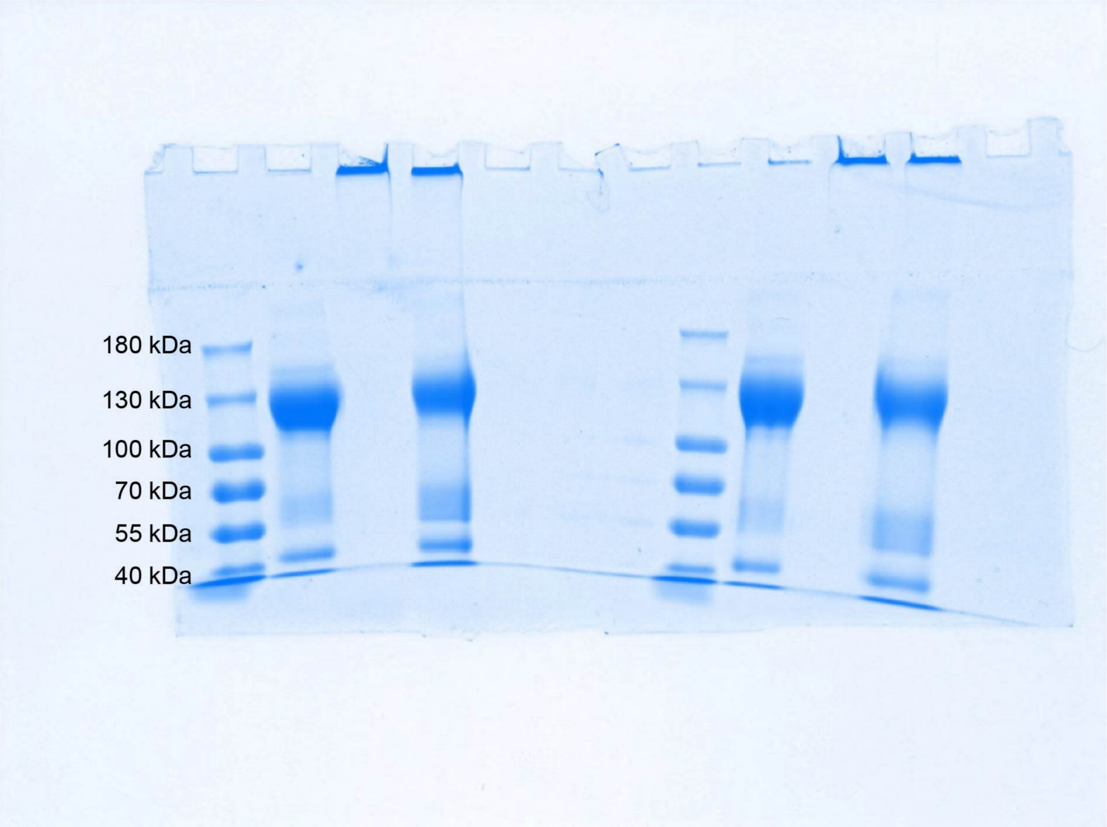

Supplementary Figure 48. Uncropped scans of gel in Supplementary Figure 8.

Case ID	WHO grade	PD-L1 IOD
LGG1	WHO II	157741.7
LGG2	WHO II	97909.9
LGG3	WHO II	99051.1
LGG4	WHO II	151023.5
LGG5	WHO II	17798.8
LGG6	WHO II	110691.3
LGG7	WHO II	124410.2
LGG8	WHO II	155358.8
LGG9	WHO II	158661.7
LGG10	WHO II	35931.6
HGG1	WHO IV	1471491.7
HGG2	WHO IV	569999.2
HGG3	WHO IV	244148.2
HGG4	WHO IV	593679.9
HGG5	WHO IV	687539.1
HGG6	WHO IV	68174.9
HGG7	WHO IV	411940.2
HGG8	WHO IV	97899.7
HGG9	WHO IV	267202.8
HGG10	WHO IV	63065.7

Supplementary Table 1. Demographic and tumor characteristics of glioma patients. LGG, low-grade glioma. HGG, high-grade glioma. PD-L1, programmed death-ligand 1. IOD, integral optical density.

	Test 1	Test 2	Test 3	Mean ± SD
IgG encapsulation efficiency (EE%)	84.595	84.993	77.354	82.31±4.30
PTX encapsulation efficiency (EE%)	90.145	86.463	86.270	87.63±2.18
IgG loading content (LC%)	23.053	23.162	21.080	22.43±1.17
PTX loading content (LC%)	49.131	47.124	47.019	47.76±1.19

Supplementary Table 2. The encapsulation efficiency and loading content of drugs in APM. The encapsulation efficiency (EE%) and loading contents (LC%) of aPD-L1 and PTX in APM. n = 3 biologically independent samples, data are presented as mean ± SD.
