# Supplementary material for: Redox-responsive polymer micelles co-encapsulating immune checkpoint inhibitors and chemotherapeutic agents for glioblastoma therapy
Source: Nat Commun. 2024 Feb 6;15:1118. doi: 10.1038/s41467-024-44963-3 (PMC10847518; doi:10.1038/s41467-024-44963-3)
Supplement: Supplementary file 3 — Reporting Summary [file 41467_2024_44963_MOESM3_ESM.pdf]

nature portfolio

Corresponding author(s): Xiaoyuan Chen, Jinbing Xie and Shenghong Ju

Last updated by author(s): Dec 15, 2023

Reporting Summary

Nature Portfolio wishes to improve the reproducibility of the work that we publish. This form provides structure for consistency and transparency in reporting. For further information on Nature Portfolio policies, see our Editorial Policies and the Editorial Policy Checklist.

Statistics

For all statistical analyses, confirm that the following items are present in the figure legend, table legend, main text, or Methods section.

n/a	Confirmed	
☐	☒	The exact sample size (*n*) for each experimental group/condition, given as a discrete number and unit of measurement
☐	☒	A statement on whether measurements were taken from distinct samples or whether the same sample was measured repeatedly
☐	☒	The statistical test(s) used AND whether they are one- or two-sided<br>*Only common tests should be described solely by name; describe more complex techniques in the Methods section.*
☐	☒	A description of all covariates tested
☐	☒	A description of any assumptions or corrections, such as tests of normality and adjustment for multiple comparisons
☐	☒	A full description of the statistical parameters including central tendency (e.g. means) or other basic estimates (e.g. regression coefficient) AND variation (e.g. standard deviation) or associated estimates of uncertainty (e.g. confidence intervals)
☐	☒	For null hypothesis testing, the test statistic (e.g. F, t, r) with confidence intervals, effect sizes, degrees of freedom and P value noted<br>*Give P values as exact values whenever suitable.*
☒	☐	For Bayesian analysis, information on the choice of priors and Markov chain Monte Carlo settings
☒	☐	For hierarchical and complex designs, identification of the appropriate level for tests and full reporting of outcomes
☒	☐	Estimates of effect sizes (e.g. Cohen's *d*, Pearson's *r*), indicating how they were calculated

Our web collection on statistics for biologists contains articles on many of the points above.

Software and code

Policy information about availability of computer code

Data collection	The size distribution and zeta potential were evaluated by dynamic light scattering measurements using a ZetaPlus (Brookhaven Instruments, New York, USA).The morphology was observed by transmission electron microscopy (TEM) (Hitachi H7650, Tokyo, Japan). The drug concentration quantified by HPLC analysis (Waters, Massachusetts, USA).The gel image was taken by Molecular Imager Gel Doc XR+ System (BioRad, California, USA). The flow-cytometry data were obtained by FACSCelesta (Becton, Dickinson and Company, New Jersey, USA) and NovoCyte Flow Cytometer (ACEA Biosciences, California, USA). A microplate reader (Multiskan Go, Thermo Fisher Scientific, Massachusetts, USA) was used to measure the absorbance at different nm. Fluorescence images were detected by Laser Scanning Confocal Microscopy (Olympus FV3000, Tokyo, Japan) and fluorescence microscope (ECLIPSE Ti2-U microscope, Nikon Corporation, Tokyo, Japan). Fluorescence of living mice were obtained by near-infrared imaging (CRi, Woburn, Massachusetts, USA). In vivo MR was performed using a 7.0-Tesla small animal MR scanner (Bruker Pharmascan, Ettlingen, Germany). Respiratory rate and body temperature were monitored using a physiological monitor (1025; SA Instruments, Stone Creek, New York). Slices were obtained under the scanner (Pannoramic MIDI, 3Dhistech, Budapest, Hungary) for image acquisition. Blood vessels were monitored by full-field laser perfusion imager (Moor Instruments Ltd, Millwey, UK)
Data analysis	All statistical analyses were performed on Graphpad Prism 8.0. SPSS 26.0 software was used for statistical analysis of all data. All flow-cytometry data were analyzed on FlowJo 10.4. For immunofluorescence,CaseViewer 2.4 was used to observe the images, and Image Pro Plus 6.0 was used to count positive cells.

For manuscripts utilizing custom algorithms or software that are central to the research but not yet described in published literature, software must be made available to editors and reviewers. We strongly encourage code deposition in a community repository (e.g. GitHub). See the Nature Portfolio guidelines for submitting code & software for further information.

Data

Policy information about availability of data

All manuscripts must include a data availability statement. This statement should provide the following information, where applicable:

- Accession codes, unique identifiers, or web links for publicly available datasets
- A description of any restrictions on data availability
- For clinical datasets or third party data, please ensure that the statement adheres to our policy

The publicly available data used in Supplementary Figure 1a, b and e are available in the TCGA database (survminer package version 0.4.9, R version 4.3.1). All data supporting the findings of this study are available within the paper and its Supplementary Information files or from the corresponding author upon reasonable request. Source data are provided with this paper.

Research involving human participants, their data, or biological material

Policy information about studies with human participants or human data. See also policy information about sex, gender (identity/presentation), and sexual orientation and race, ethnicity and racism.

Reporting on sex and gender	11 female, 7 male involved in the study and sex and/or gender information of 2 other were not been collected. Sex and gender were not considered in study design. Sex and/or gender was determined based on self-reporting. We have removed that are not necessary to support the scientific claims from the manuscript that individuals can no longer be identified.
Reporting on race, ethnicity, or other socially relevant groupings	n/a
Population characteristics	3 patients aged 20-35, 5 patients aged 35-50, 6 patients aged 50-65, 1 patient was older than 65, and age information of 5 other were not been collected.
Recruitment	n/a
Ethics oversight	All procedures performed in studies involving human participants were in accordance with the ethical standards of Ethics Review Committee of Huashan Hospital Affiliated to Fudan University and the Independent Ethics Committee for Clinical Research of Zhongda Hospital, Affiliated to Southeast University.

Note that full information on the approval of the study protocol must also be provided in the manuscript.

Field-specific reporting

Please select the one below that is the best fit for your research. If you are not sure, read the appropriate sections before making your selection.

☒ Life sciences ☐ Behavioural & social sciences ☐ Ecological, evolutionary & environmental sciences

For a reference copy of the document with all sections, see nature.com/documents/nr-reporting-summary-flat.pdf

Life sciences study design

All studies must disclose on these points even when the disclosure is negative.

Sample size	Sample sizes were determined according to a pilot study as well as on the basis of previous experimental experience.
Data exclusions	No data were excluded.
Replication	All attempts at replication were successful.
Randomization	All samples and organisms were randomly allocated into the experimental groups.
Blinding	The investigators were blinded to group allocation during data colection and/or analysis.

Reporting for specific materials, systems and methods

We require information from authors about some types of materials, experimental systems and methods used in many studies. Here, indicate whether each material, system or method listed is relevant to your study. If you are not sure if a list item applies to your research, read the appropriate section before selecting a response.

Materials & experimental systems

n/a	Involved in the study
☐	☒ Antibodies
☐	☒ Eukaryotic cell lines
☒	☐ Palaeontology and archaeology
☐	☒ Animals and other organisms
☐	☒ Clinical data
☒	☐ Dual use research of concern
☒	☐ Plants

Methods

n/a	Involved in the study
☒	☐ ChIP-seq
☐	☒ Flow cytometry
☒	☐ MRI-based neuroimaging

Antibodies

Antibodies used

Anti-mouse PD-L1 (B7-H1) (Cat. No. BE0101), anti-CD4 (Cat. No. BE0119), anti-CD8 (Cat. No. BE0061) was purchased from Bio X Cell (New Hampshire, USA). Rabbit IgG control (whole molecule), purified (Cat. No. A01008) was purchased from GenScript (Jiangsu, China). CD16/CD32 monoclonal antibody (Cat. No. 14-0161-81) and the antibodies used for flow cytometry were specific for CD3 (Cat. No. 69-0032-82), CD4 (Cat. No. 11-0041-82), CD8a (Cat. No. 78-0081-82), CD25 (Cat. No. 17-0251-81), CD127 (Cat. No. 12-1271-82), CD11c (Cat. No. 45-0114-82), MHC ? (Cat. No. 64-5321-82), CD80 (Cat. No. 62-0801-82), CD86 (Cat. No. 63-0862-80), CD133 (Cat. No.11-1331-80), CD44 (Cat. No.45-0441-80), NESTIN (Cat. No. MA523574), SOX2 (Cat. No. 50-9811-80), F4/80 (Cat. No. 12-4801-82), PD-L1 (Cat. No. 14-5983-82), PD-1(14-278-82), CD11b (MA1-80091) were purchased from eBioscience (California, USA). The antibodies used for flow cytometry were specific for CD44 (Cat. No. 103049) and CD62L (Cat. No. 104407), CD45 (Cat. No. 1447705), PD-1 (Cat. No. 114117), TIM-3 (Cat. No. 119723), NK1.1 (Cat. No. 108745), Gr-1(Cat. No. 108443), Ly6C (Cat. No. 128032), Ly6G (Cat. No. 127614), Granzyme B (Cat. No. 396424), IFN-? (Cat. No. 505810), CD11b (Cat. No. 101206), CD206 (Cat. No. 321124) were purchased from Biolegend (California, USA). anti-mouse Calreticulin (CRT) monoclonal antibody was purchased from Cell Signaling Technology (Cat. No. 12238s), anti-human CRT (Cat. No. 10292-1-AP) and anti-human TIM-3 (Cat. No. 60355-1-Ig) monoclonal antibody was purchased from Proteintech. HMGB1 monoclonal antibody was purchased from Abcam (Cat. No. ab18256). The antibodies used for immunofluorescence were specific for CD3 (Cat. No. GB13014), CD4 (Cat. No. GB13064-2), CD8 (Cat. No. GB13429), Foxp3 (Cat. No. GB112325), CD11c (Cat. No. GB11059), F4/80 (Cat. No. GB13373), CD206 (Cat. No. GB13438), iNOS (Cat. No. GB11119), Arg1 (Cat. No. GB11285) were purchased from Wuhan Servicebio Technology Co., Ltd (Hubei, China). The secondary antibodies used for immunostaining were FITC conjugated goat anti-rat IgG (H&L) (Cat. No. SA00003-11, Proteintech Group Inc., Illinois, USA), Cy3 conjugated goat anti-rat IgG (H&L) (Cat. No. SA00009-1, Proteintech Group Inc., Illinois, USA), goat anti-rabbit IgG H&L (Alexa Fluor 647) (Cat. No. ab150079, Abcam, Cambridge, UK), HRP conjugated goat anti-rabbit IgG (H&L) (Cat. No. GB23303, Servicebio, Hubei, China), Cy3 conjugated goat anti-rabbit IgG (H&L) (Cat. No. GB21303, Servicebio, Hubei, China) and Alexa Fluor 549 conjugated goat anti-rabbit IgG (H&L) (Cat. No.111-585-003, Jackson ImmunoResearch Inc., Pennsylvania, USA). Enzyme-linked immunosorbent assay (ELISA) kits were used to measure the culture medium concentration of IgG (Cat. No. CSB-E06949Rb, Cusa Bio., Hubei, China) and HMGB1 (Cat. No. ARG81310, Arigo Biolaboratories Co., Taiwan, China).

Validation

The validation of each antibody was done according to the standard information offered by the supplier.

Eukaryotic cell lines

Policy information about cell lines and Sex and Gender in Research

Cell line source(s)

The murine GL261 cells and bEnd.3 cells were obtained from KeyGEN Biotech. Co., Ltd. (Jiangsu, China). The murine G422 cells was obtained from FuHeng Biology (Shanghai, China). The G7 and WL1 patient-derived xenograft (PDX) cell lines were obtained from Professor Xiuxing Wang's laboratory cell bank. The human U87 cells and U251 cells and the murine GL261-GFP and G422-GFP cells were obtained from our laboratory cell bank.

Authentication

None of the cell lines used were authenticated.

Mycoplasma contamination

The cell lines were not tested for mycoplasma contamination.

Commonly misidentified lines (See ICLAC register)

No commonly misidentified cell lines were used.

Animals and other research organisms

Policy information about studies involving animals; ARRIVE guidelines recommended for reporting animal research, and Sex and Gender in Research

Laboratory animals

C57BL/6 mice (male; 6-8 weeks old), Kunming mice (female; 4-5 weeks old), BALB/c nu mice (female; 6-8 weeks old) and SD rats (male; 6-8 weeks old) were purchased from Cavens Biogle(suzhou) Model Animal Research Co., Ltd. (Jiangsu, China).

Wild animals

The study did not involve wild animals.

Reporting on sex

Gender preference analysis was not performed for ethical reasons and the study is gender-neutral.

Field-collected samples

The study did not involve samples collected from the field.

Ethics oversight	All animal experiments were conducted according to the ethical guidelines of the Animal Care & Welfare Committee of Southeast University, Jiangsu, China.

Note that full information on the approval of the study protocol must also be provided in the manuscript.

Clinical data

Policy information about clinical studies

All manuscripts should comply with the ICMJE guidelines for publication of clinical research and a completed CONSORT checklist must be included with all submissions.

Clinical trial registration	n/a
Study protocol	Brain tumor specimens were obtained from patients with informed consent and were reviewed by the pathologist and surgeon. Pathologist classified the type and grade of the tumors in accordance with the WHO histological grading of central nervous system tumors.
Data collection	14 cases of gliomas were selected from Huashan Hospital, Fudan University, and 6 cases of gliomas were selected from Zhongda Hospital, Southeast University.
Outcomes	We evaluated the integral optical density (IOD) of PD-L1 immunofluorescence (IF) in the samples to quantify the PD-L1 expression (Supplementary Fig. 1c and d, Table S1). The PD-L1 was considerably higher in the HGG group than in LGG groups ($P < 0.05$).

Flow Cytometry

Plots

Confirm that:

☒ The axis labels state the marker and fluorochrome used (e.g. CD4-FITC).

☒ The axis scales are clearly visible. Include numbers along axes only for bottom left plot of group (a 'group' is an analysis of identical markers).

☒ All plots are contour plots with outliers or pseudocolor plots.

☒ A numerical value for number of cells or percentage (with statistics) is provided.

Methodology

Sample preparation	For the flow cytometry analysis of the tumor immuno-microenvironment, tumor tissue was removed 3 days after the last injection of different treatments (1.5 or 3 mg/kg on an aPD-L1 or PTX basis). For T cell analysis, mice lymphocyte separation medium was used for dissociation. After sorting with anti-CD45 magnetic beads, the tumor cells were stained with anti-CD3, anti-CD8, anti-CD4, anti-CD25, and anti-CD127 antibodies for 30 minutes. For DC analysis, mice lymphocyte separation medium was used for dissociation. After sorting with anti-CD45 magnetic beads, the tumor cells were stained with anti-CD11c, anti-MHC ?, anti-CD80, and anti-CD86 antibodies for 30 minutes.For the CRT expression, cell suspensions were incubated with anti-CRT polyclonal antibody (1:100 dilution) for 1 h at 37 °C. Then, cells were washed with 1% BSA buffer twice and stained with Alexa Fluor 647 labeled goat antirabbit IgG (H&L) highly cross-adsorbed secondary antibody (1:200 dilution) for 30 min at 4 °C. To analyze effector memory T cells, mice treated with A2-APM were selected at day 45 post-inoculation. Brain, dLNs and spleen were removed and stained with anti-CD3, anti-CD8, anti-CD44 and anti-CD62L antibodies.
Instrument	Flow cytometry (NovoCyte Flow Cytometer, ACEA Biosciences, California, USA) flow cytometry (FACSCelesta, Becton,Dickinson and Company, New Jersey, USA)
Software	FlowJo10.4
Cell population abundance	Mice lymphocyte separation medium was used for dissociation, and sorted with anti-CD45 magnetic beads (collecting 10,000 events for analysis).
Gating strategy	The boundaries of positive and negative staining cell populations are distinguished by microsphere single staining tubes. All gating strategies were described in supplementary information.

☒ Tick this box to confirm that a figure exemplifying the gating strategy is provided in the Supplementary Information.
