# Supplementary material for: Redox-responsive polymer micelles co-encapsulating immune checkpoint inhibitors and chemotherapeutic agents for glioblastoma therapy
Source: Nat Commun. 2024 Feb 6;15:1118. doi: 10.1038/s41467-024-44963-3 (PMC10847518; doi:10.1038/s41467-024-44963-3)
Supplement: Supplementary file 4 — Source Data [file 41467_2024_44963_MOESM4_ESM.zip › ALL Source Data/Source data for Figure 3j.pdf]

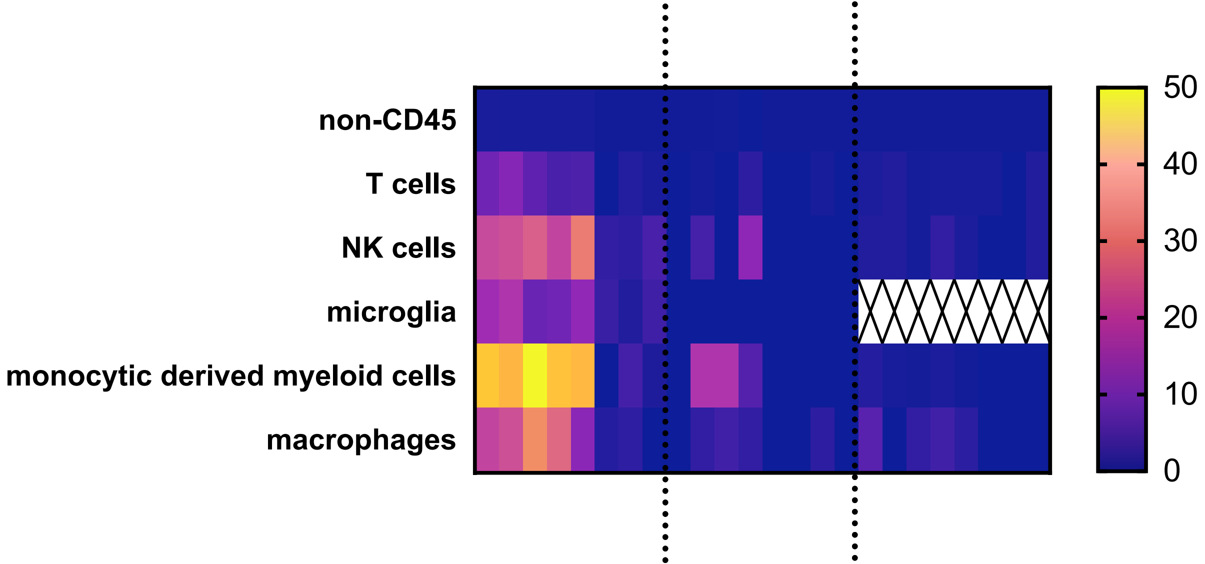
non-CD45
T cells
NK cells
microglia
monocytic derived myeloid cells
macrophages
50
40
30
20
10
0
