# Supplementary material for: Redox-responsive polymer micelles co-encapsulating immune checkpoint inhibitors and chemotherapeutic agents for glioblastoma therapy
Source: Nat Commun. 2024 Feb 6;15:1118. doi: 10.1038/s41467-024-44963-3 (PMC10847518; doi:10.1038/s41467-024-44963-3)
Supplement: Supplementary file 4 — Source Data [file 41467_2024_44963_MOESM4_ESM.zip › ALL Source Data/Source data for HMGB1/Source data for HMGB1 fluorescence images.zip!/Source data for HMGB1 fluorescence images/Description for the source data of HMGB1.docx]

Description of source data

The images are named "group-merge-repeat times", this experiment was conducted in three separate experiments at different times, so the image size and cell states are slightly different.
